# Neuronal AIMP2–α-synuclein synergy drives endogenous tau and amyloid-β pathologies and neurodegeneration in a mechanistic model of Lewy body dementia

**DOI:** 10.1038/s41419-026-09181-3

**Published:** 2026-08-13

**Authors:** Doeun Kim, Heejeong Kim, Ji Hun Kim, Hyojung Kim, Joo-Ho Shin, Han Seok Ko, Eunyoung Moon, Hee-Seok Kweon, Yunjong Lee

**Affiliations:** 1 Department of Pharmacology, Samsung Biomedical Research Institute, Sungkyunkwan University School of Medicine, Suwon, Republic of Korea; 2 Department of Neurology, Johns Hopkins University School of Medicine, Baltimore, MD USA; 3 Center for Bio-imaging & Translational Research, Korea Basic Science Institute, Cheongju, Republic of Korea

**Keywords:** Parkinson's disease, Dementia

## Abstract

Lewy body dementia (LBD), encompassing Parkinson’s disease dementia (PDD) and dementia with Lewy bodies (DLB), is defined by widespread α-synuclein (αSyn) aggregation and frequently exhibits coexistent tau and amyloid-β (Aβ) pathologies. However, whether αSyn pathology is sufficient to drive endogenous tau and Aβ aggregation has remained unclear due to a lack of in vivo models that recapitulate the full spectrum of LBD-associated proteinopathies and neurodegeneration. AIMP2, a parkin substrate that accumulates in Parkinson’s disease, enhances αSyn aggregation and toxicity, but its role in initiating downstream mixed pathologies across vulnerable brain regions has not been demonstrated in vivo. Here, we generated a conditional, neuron-specific Tet-Off double-transgenic mouse model enabling post-developmental coexpression of AIMP2 and A53T αSyn. This synergistic coexpression induced rapid and widespread αSyn aggregation, detergent-insoluble Lewy-like inclusions, mitochondrial degeneration, synaptic impairment, and neuronal loss, accompanied by marked gliosis. Mice developed key clinical features of LBD, including progressive cognitive impairment, bradykinesia, and olfactory deficits. Notably, without tau or APP overexpression, endogenous tau became hyperphosphorylated through dysregulated kinase signaling, and Aβ accumulated predominantly as TBS-soluble Aβ42 monomers/oligomers without fibrillar plaque deposition, demonstrating that AIMP2–αSyn–driven pathology is sufficient to trigger downstream tauopathy and Aβ pathologies. These mixed pathologies were associated with lysosomal and proteasomal dysfunction, evidenced by p62 and polyubiquitinated protein accumulation. Proteomic analysis identified dysregulation of pathways linked to amyloid processing, synaptic trafficking, and protein quality control. Importantly, temporal suppression of AIMP2 and αSyn expression promoted reduction of pre-existing pathologies and partially restored cognitive function. This study establishes a genetically controlled in vivo model of LBD-like mixed proteinopathy recapitulating αSyn aggregation, tau hyperphosphorylation, Aβ accumulation, neurodegeneration, and behavioral decline. These findings support a mechanistic link between AIMP2-enhanced αSyn toxicity and secondary proteinopathies and position this model as a platform for mechanistic and therapeutic studies in complex α-synucleinopathies.

**Figure:**
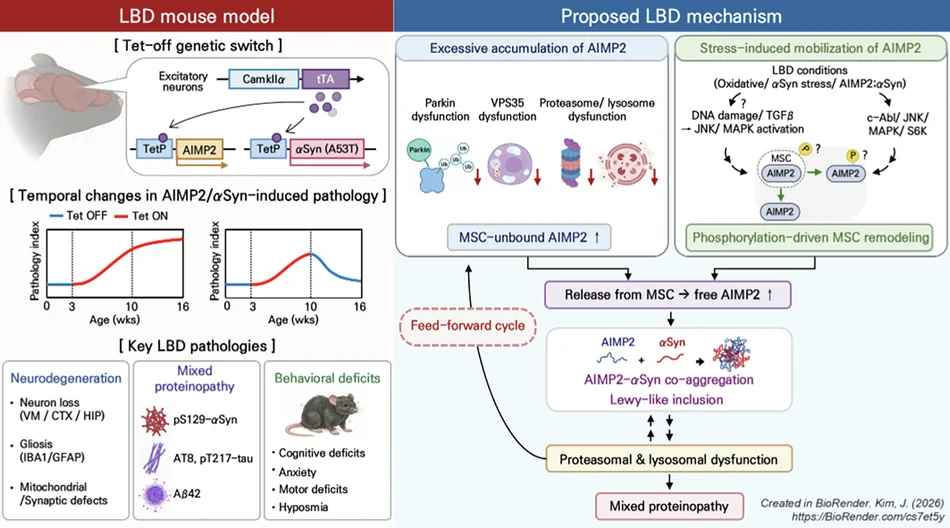

## Introduction

Lewy body dementia (LBD)—encompassing dementia with Lewy bodies (DLB) and Parkinson’s disease dementia (PDD)—is the second most common neurodegenerative dementia after Alzheimer’s disease (AD) [1]. LBD is defined by widespread α-synuclein (αSyn) accumulation in Lewy bodies and Lewy neurites and frequently shows co-pathology with amyloid-β (Aβ) plaques and hyperphosphorylated tau tangles, lesions classically linked to AD [2]. Whether αSyn pathology mechanistically drives tau and Aβ accumulation, or whether these proteinopathies arise independently within shared pathogenic milieus, remains unresolved. Clarifying this interplay is essential for understanding LBD progression and developing disease-modifying therapies.

Existing models only partially capture LBD. Neuronal αSyn overexpression (e.g., PrP, Thy1 promoters) produces severe brainstem/spinal neurodegeneration and late paralysis but limited early cortical/hippocampal pathology [3]. Preformed fibril (PFF) models recapitulate prion-like spread and mixed symptoms [4], yet require invasive injections, suffer batch variability, and often need >6 months for prominent phenotypes. Thus, there is a need for genetically controlled, rapid-onset models that develop broad regional pathology, mixed proteinopathies, and behaviorally relevant deficits—and that are suitable for unbiased proteomics.

AIMP2 (ARS-interacting multifunctional protein 2), a Parkin substrate, accumulates in PD brains and promotes αSyn aggregation via direct interaction [5–7]. Viral co-expression in substantia nigra enhances Lewy-like pathology and dopaminergic toxicity [5], but whether this synergy extends to cortex, hippocampus, and olfactory bulb is unknown. Tet-Off models individually expressing AIMP2 [8] or αSyn [9] demonstrate neurotoxicity, yet neither alone yields widespread Lewy-like inclusions, and developmental αSyn overexpression can cause nonspecific hippocampal toxicity [10]. A conditional system enabling post-developmental, neuron-specific co-expression of AIMP2 and αSyn could therefore interrogate combined pathogenic effects while minimizing developmental confounds.

Here we generate Tet-Off double-transgenic mice with neuron-specific, post-developmental co-expression of AIMP2 and A53T αSyn. These mice rapidly develop widespread Lewy-like inclusions, detergent-insoluble co-aggregation of AIMP2/αSyn, mitochondrial and synaptic ultrastructural abnormalities, substantial neurodegeneration with gliosis, and marked nigrostriatal dopaminergic loss. Behaviorally, they exhibit cognitive, motor (bradykinesia/coordination), and olfactory deficits mirroring LBD. Unbiased proteomics reveals dysregulation of Aβ and tau-related pathways, and we detect Aβ accumulation and hyperphosphorylated tau in affected regions. This model provides a platform to study AIMP2–αSyn interaction and mixed proteinopathies underlying LBD.

## Results

### Generation of conditional double-transgenic (Tg) mice expressing AIMP2 and α-synuclein (αSyn)

**Fig. 1:**
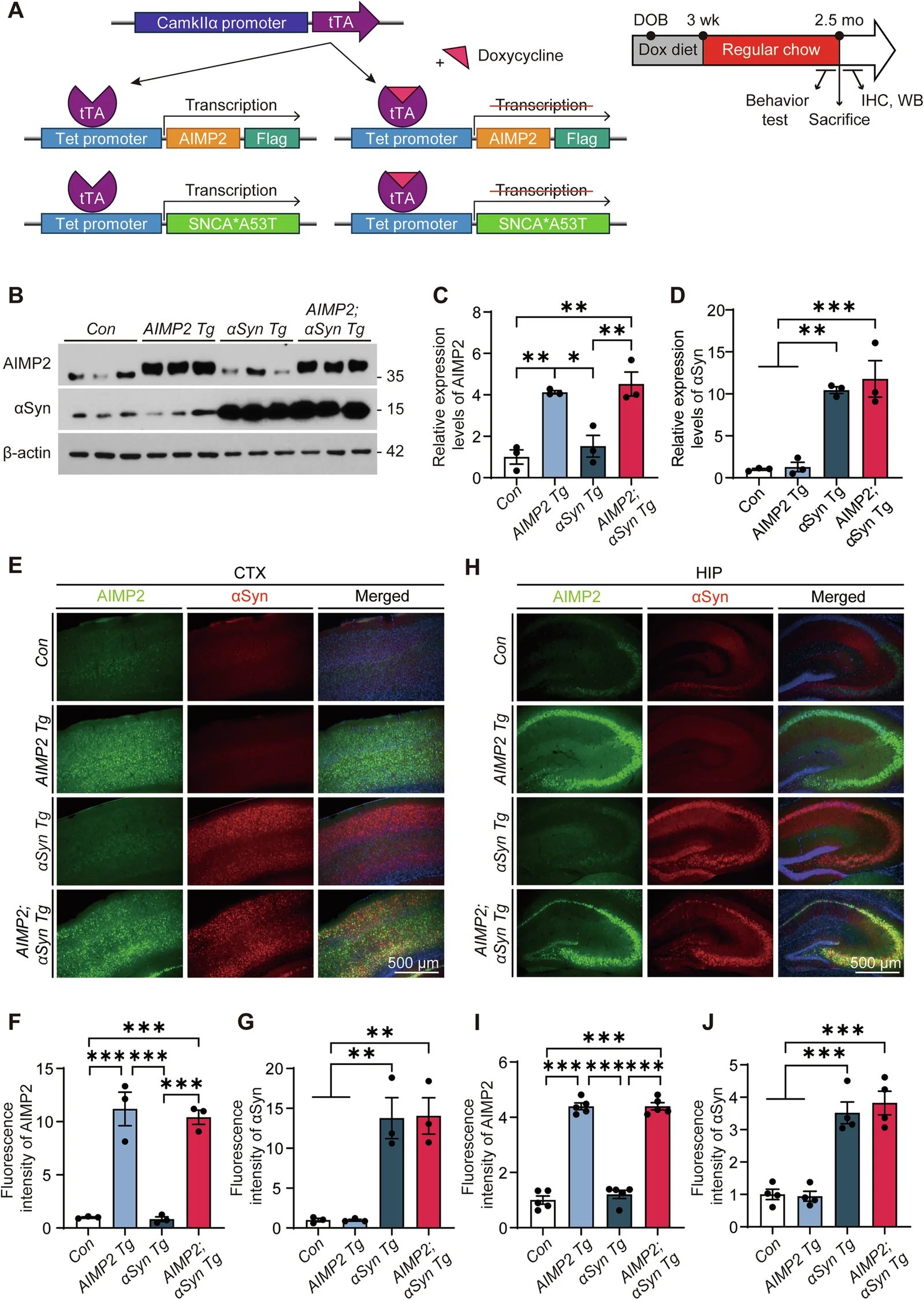
Generation of conditional transgenic (Tg) mice coexpressing AIMP2 and A53T-α-synuclein (αSyn) in mouse brains. **A** Schematic summary of Tet-OFF conditional expression of AIMP2 and αSyn (A53T) driven by tTA binding to Tet promoter. tTA expression is induced by pan-neuronal promoter CamKIIα. tTA is inactivated by doxycycline binding, turning off AIMP2 and αSyn expression (left panel). Experimental schedule and transgene expression protocol in which AIMP2 and αSyn expression is induced at 3 weeks of age by switching from doxycycline diet to regular chow (right panel). IHC immunohistochemistry, WB western blot. **B** Representative immunoblots of αSyn and AIMP2 in the whole brain (except for cerebellum) lysates from the indicated genotypes at 2.5 months of age. β-actin serves as an internal loading control. **C**, **D** Quantification of relative expression of AIMP2 and αSyn in the brains from the indicated mouse groups normalized to β-actin (*n* = 3 mice per group). **E** Representative immunofluorescence images of AIMP2 and αSyn in the cortex (CTX) coronal sections from the indicated 2.5 month old mouse groups. **F**, **G**) Quantification of relative immunofluorescence signals of AIMP2 and αSyn in the cortex from the indicated mouse groups (*n* = 3 mice per group). **H** Representative immunofluorescence images of AIMP2 and αSyn in the hippocampal (HIP) sections from the indicated 2.5 month old mouse groups. **I**, **J** Quantification of relative immunofluorescence signals of AIMP2 (*n* = 5 mice per group) and αSyn (*n* = 4 mice per group) in the hippocampus from the indicated mouse groups. Data are expressed as mean ± SEM. Statistical analysis was performed using one-way analysis of variance (ANOVA) test followed by Tukey’s post-hoc analysis. *p < 0.05, **p < 0.01, and ***p < 0.001. The uncropped Western blot panels are provided in Suppl. fig. 10.

We used the Tet-Off system: CamKIIα-tTA driver mice were crossed with TetP-AIMP2;TetP-αSyn (A53T) responders (Suppl. Fig. 1A). In CamKIIα-tTA; TetP-AIMP2; TetP-αSyn mice (hereafter AIMP2;αSyn), neuronal transgene expression is tTA-dependent and suppressed by doxycycline (Dox). Dox feeding during breeding and until postnatal week 3 prevented developmental expression; switching to regular chow induced brain expression of AIMP2 and/or αSyn by immunoblot (Fig. 1B–D). Immunofluorescence confirmed elevated expression in cortex and hippocampus—regions with high CamKIIα activity [11, 12]—across single- and double-Tg lines, with extensive overlap in AIMP2;αSyn mice (Fig. 1E–J). Immunohistochemistry revealed accumulation of both proteins in olfactory bulb, striatum, cortex, hippocampus, and ventral midbrain of AIMP2;αSyn mice versus littermate controls (Suppl. Fig. 1C, D). Thus, neuronal AIMP2/αSyn co-expression is tightly controlled and inducible/off-switchable by Dox.

### Rapid Lewy-like inclusion formation and ultrastructural deficits

**Fig. 2:**
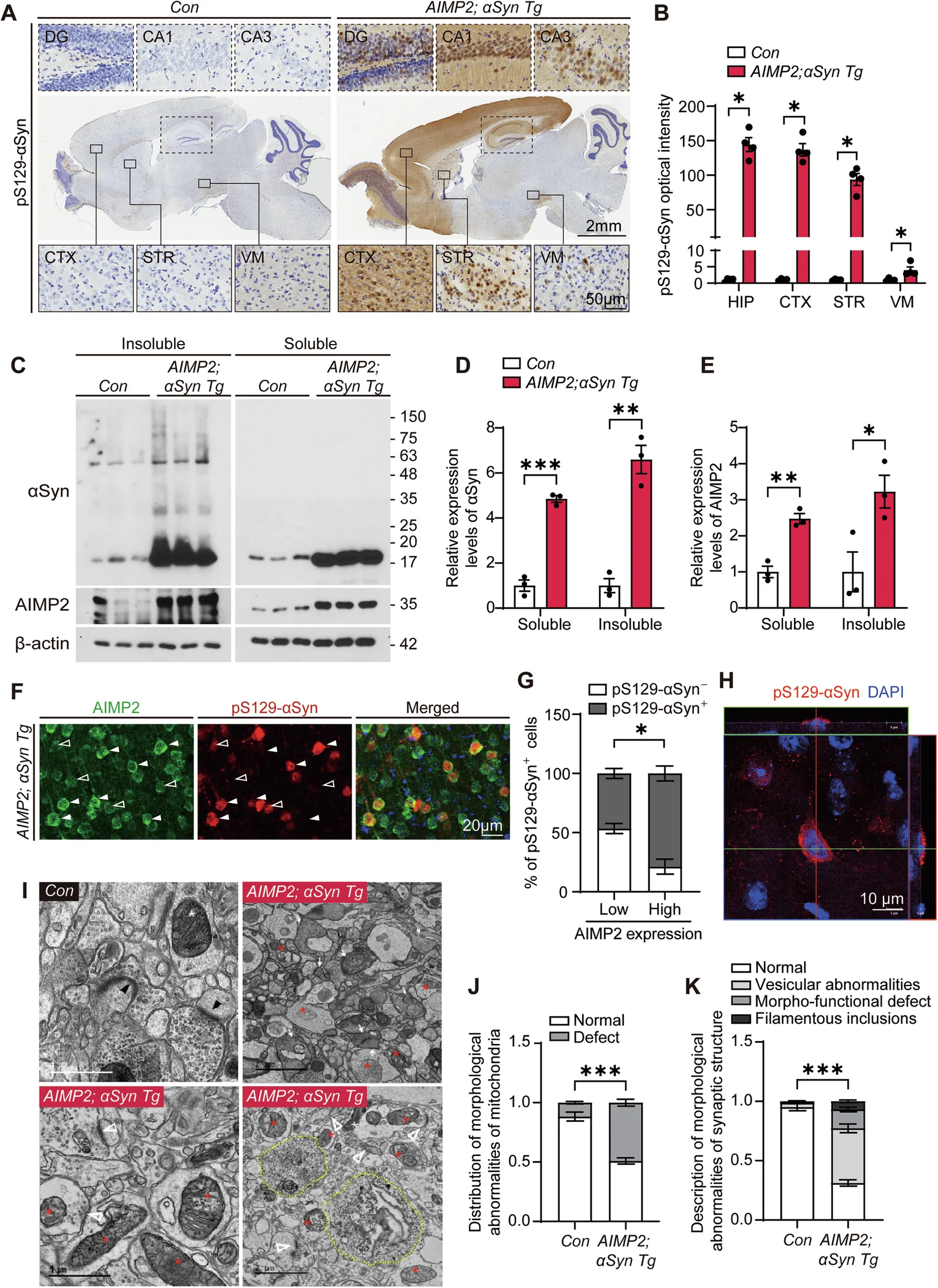
Coexpression of AIMP2 and αSyn leads to the formation of Lewy-like inclusion and mitochondria impairment. **A** Representative immunohistochemistry images of αSyn aggregates in the sagittal sections from 2.5 month-old AIMP2;αSyn Tg and littermate CamKIIα-tTA (Con) mice using pS129-αSyn specific antibody. The sections were counterstained with Nissl. Magnified images showing pS129-αSyn signal for the selected brain subregions are also presented. Scale bar = 50 μm, and 2 mm as indicated. CTX, cortex; DG, dentate gyrus; STR, striatum; VM, ventral midbrain. **B** Quantification of pS129-αSyn signals in the cortex, hippocampus, striatum and ventral midbrains from AIMP2;αSyn Tg and littermate control mice (*n* = 4 mice per group). (**C**) Expression and distribution of αSyn and AIMP2 in the detergent soluble and insoluble fractions prepared from brains of 2.5 month-old AIMP2;αSyn Tg and littermate control mice monitored by western blot using the indicated specific antibodies. β-actin serves as internal loading control for each sample. **D**, **E** Quantification of relative αSyn and AIMP2 expression in the detergent soluble and insoluble fractions prepared from the indicated mouse groups (*n* = 3 mice per group). **F** Co-immunofluorescence images showing distribution of AIMP2 and pS129-αSyn in the cortex of AIMP2;αSyn Tg mice. Scale bar = 20 μm. Cells with low and high AIMP2 immunofluorescence signals are indicated by empty and filled arrowheads, respectively. **G** Quantification of the percentage of pS129-αSyn–positive cells in groups with low versus high AIMP2 signal intensities. (*n* = 206 cells from 4 mice per group). **H** Confocal immunofluorescence image with z-stack showing perinuclear distribution of pS129-αSyn–positive Lewy-like inclusions in cortical cells of AIMP2/αSyn Tg mice. Nuclei were counterstained with DAPI. **I** Representative electron micrographs of cortex sections from 2.5-month-old AIMP2;αSyn Tg mice and littermate controls. Normal and abnormal mitochondria are indicated by white and red asterisks, respectively. Black arrowheads denote intact synaptic terminals, while white arrowheads mark synaptic terminals exhibiting structural defects. Arrows indicate electron-dense synaptic terminals with structural abnormalities. Lewy-like inclusions are indicated by yellow dashed lines. **J** Percent ratio of mitochondria with normal vs. defective ultrastructural morphology in control and AIMP2;αSyn Tg mice (*n* = 35 images from 3 mice per group). **K** Ultrastructural analysis of synapses in control and AIMP2;αSyn Tg mice. The ratio of normal to defective synapses was calculated for control and AIMP2;αSyn Tg mice (*n* = 35 images from 3 mice per group). Defective synapses were characterized by irregularly shaped vesicles that were densely distributed throughout the presynaptic terminals. (vesicular abnormalities), structural deformation or internal damage (morpho-functional defect), and inclusion bodies containing filaments and tubules (filamentous inclusions). Data are expressed as mean ± SEM. Statistical analysis was performed using the Mann–Whitney test (**B**, **G**, **J**, **K**) and the two-tailed Student’s t-test (**D**, **E**). *p < 0.05, **p < 0.01, and ***p < 0.001. The uncropped Western blot panels are provided in Suppl. fig. 10.

Given prior evidence that AIMP2 accelerates αSyn aggregation in dopaminergic neurons [5], we examined pS129- αSyn pathology 2.5 months after induction (starting at week 3). Control and AIMP2-only mice lacked detectable pS129-αSyn (Fig. 2A, B; Suppl. Fig. 2A). αSyn-only mice showed modest hippocampal pS129-αSyn, whereas AIMP2;αSyn mice exhibited widespread pS129-αSyn-positive aggregates in cortex and hippocampus, and in olfactory bulb and striatum with lower signal in ventral midbrain and hypothalamus (Fig. 2A, B; Suppl. Fig. 2B, C). AIMP2-accelerated αSyn pathology was absent in GAD67-positive hippocampal interneurons, consistent with the lack of CamKIIα-tTA activity in inhibitory neurons [13] (Suppl. Fig. 2D). Thus, broad pS129-αSyn pathology emerges within ~2 months across multiple regions.

Biochemical fractionation showed increased detergent-insoluble αSyn, including high-molecular-weight species, in AIMP2;αSyn brains versus controls, with concurrent redistribution of AIMP2 to insoluble fractions (Fig. 2C–E). At the cellular level, neurons with higher AIMP2 expression displayed stronger pS129-αSyn signals (Fig. 2F, G). Z-stack confocal imaging localized Lewy-like inclusions to perinuclear/nuclear compartments and processes (Fig. 2H; Suppl. Fig. 2E). Some pS129-αSyn inclusions co-localized with AIMP2, although many neurons showed non-overlapping distributions (Suppl. Fig. 2F, G), suggesting both direct and indirect relationships between AIMP2 and αSyn in aggregate formation.

Transmission electron microscopy demonstrated increased numbers of morphologically defective mitochondria with fragmented/disorganized cristae in AIMP2;αSyn cortex compared with controls (Fig. 2I, J). Synaptic abnormalities included irregularly shaped, densely packed presynaptic vesicles, structural deformations, and inclusion bodies containing fibrillar aggregates/membranous fragments (Fig. 2I, K; Suppl. Fig. 2H). Thus, neuronal AIMP2/αSyn co-expression rapidly induces widespread Lewy-like inclusions accompanied by mitochondrial and synaptic structural impairments.

### Cortical/hippocampal neurodegeneration, neuroinflammation, and nigrostriatal loss

**Fig. 3:**
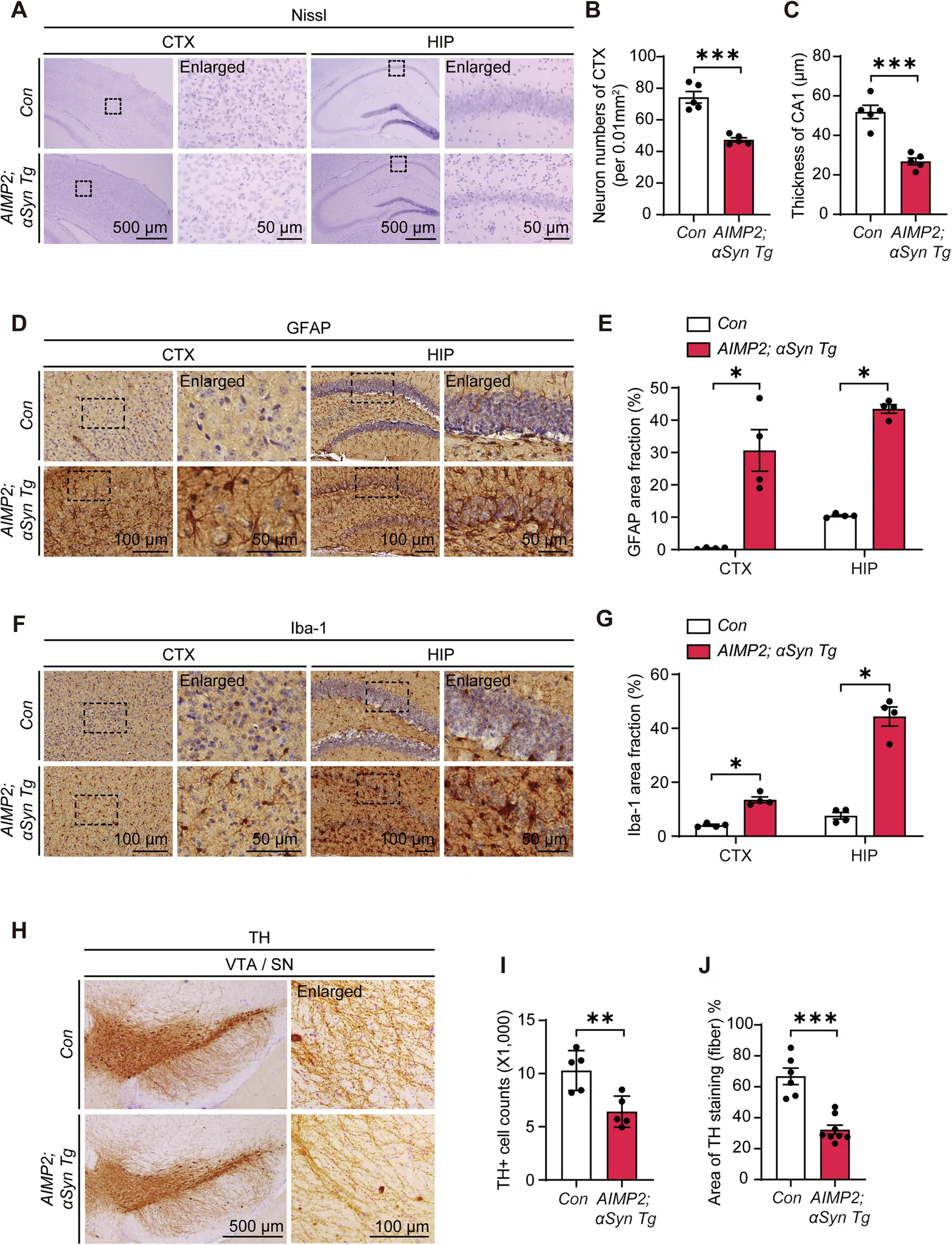
AIMP2/αSyn coexpression in mouse brains causes neurodegeneration and inflammation. **A** Representative Nissl stained cortex and hippocampus coronal sections from 2.5 month old control and AIMP2;αSyn Tg mice. The area marked with square with dotted line were magnified and shown as the enlarged images. Scale bar = 500, and 50 μm as indicated in the images. **B** Quantification of Nissl stained neuron numbers in the cortex of 2.5 month old control and AIMP2;αSyn Tg mice (*n* = 5 mice per group). **C** Thickness of CA1 of hippocampus for 2.5 month old control and AIMP2;αSyn Tg mice (*n* = 5 mice per group). **D** Anti-GFAP immunohistochemistry images of cortex and hippocampus regions from 2.5 month old control and AIMP2;αSyn Tg mice. Scale bar = 100 μm, and 50 μm, as indicated in the images. **E** % area fraction of GFAP signals in the cortex and hippocampus area of the indicated mouse groups (*n* = 4 mice per group). **F** Anti-Iba-1 immunohistochemistry images in the cortex and hippocampus coronal brain sections from 2.5 month old control and AIMP2;αSyn Tg mice. Enlarged images are also presented in the bottom panel. Scale bar = 100, 50 μm as indicated in the images. **G** % area fraction of Iba-1 signals in the cortex and hippocampus area of the indicated mouse groups (*n* = 4 mice per group). **H** Representative images of dopamine neurons in the ventral midbrain and magnified images of dopaminergic processes in the substantia nigra reticularis of 2.5 month old control and AIMP2;αSyn Tg mice as monitored by anti-TH immunohistochemistry. Scale bar = 500, 100 μm as indicated in images. **I** Stereological counting of TH stained dopamine neurons in the substantia nigra pars compacta of each mouse group (*n* = 5 mice per group). **J** Quantification of dopaminergic processes densities in the substantia nigra reticularis of each mouse group as determined by % area fraction of TH positive fibers (*n* = 6 Con, 8 AIMP2;αSyn Tg). Data are expressed as mean ± SEM. Statistical analysis was performed using the Mann–Whitney test (**E**, **G**) and the two-tailed Student’s t-test (**B**, **C**, **I**, **J**). *p < 0.05, **p < 0.01, and ***p < 0.001.

Stereological analyses revealed marked cortical neuronal loss (Nissl) and reduced CA1 thickness in AIMP2;αSyn mice versus controls (Fig. 3A–C). GFAP and Iba1 immunoreactivity were significantly increased in cortex and hippocampus, indicating neuroinflammation (Fig. 3D–G). Colabeling of pS129-αSyn and Iba-1 in the cortex and hippocampal DG revealed increased microgliosis correlating with αSyn pathology, along with marked microglial infiltration into neuron-dense DG regions in AIMP2;αSyn mice (Suppl. Fig. 3A–E). Given Lewy-like inclusions in ventral midbrain/striatum, we assessed dopaminergic integrity. Unbiased stereology showed ~37.5% loss of TH-positive nigral neurons at 2.5 months post-induction, with reduced TH fibers in substantia nigra reticulata (Fig. 3H–J). Hence, co-expression precipitates rapid degeneration and inflammation across cortex, hippocampus, and dopaminergic midbrain.

### LBD-relevant behavioral impairments

**Fig. 4:**
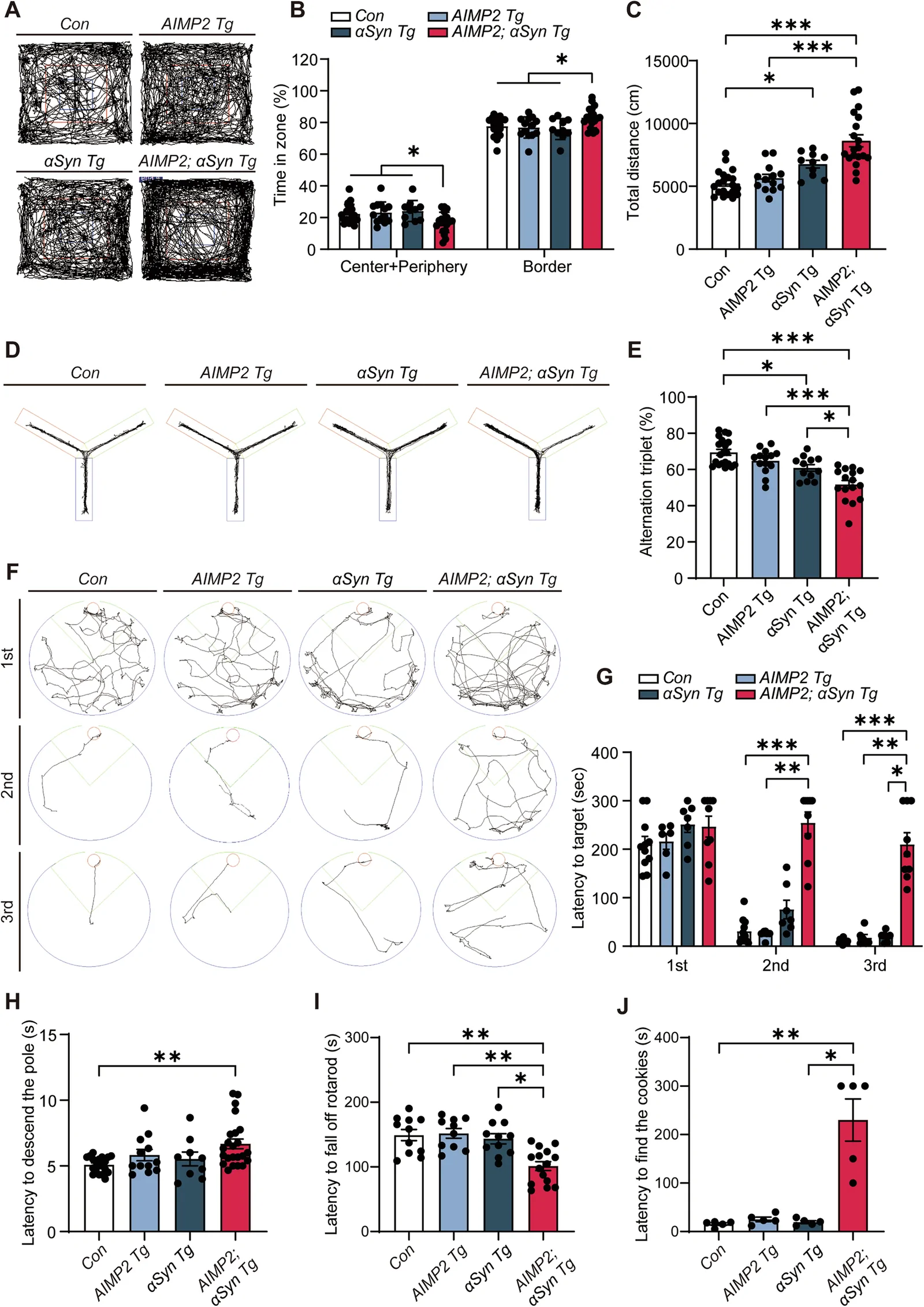
AIMP2/αSyn Tg mice develop cognitive, motor, and olfactory impairments. **A** Representative exploratory paths of 2.5 month old mice with the indicated genotypes in an open field arena. **B** % time spent in the designated regions (border vs. center+periphery) by the indicated mouse groups (*n* = 20 per Con, 13 per AIMP2 Tg, 10 per αSyn Tg, 19 per AIMP2;αSyn Tg mice). **C** Total distance traveled by each mouse group in an open filed test session (*n* = 20 per Con, 13 per AIMP2 Tg, 10 per αSyn Tg, 19 per AIMP2;αSyn Tg mice). **D** Representative travel paths of the indicated mouse groups in the Y-maze with three arms. **E** % spontaneous alteration by each mouse group in the Y-maze (*n* = 20 per Con, 14 per AIMP2 Tg, 12 per αSyn Tg, 16 per AIMP2;αSyn Tg mice). **F** Representative travel paths by each mouse group in three trials (1st-trial on day 1; 2nd-trial on day 3; 3rd-trial on day 8) of Barnes maze. The location of the hole (hiding cage underneath) in the circular platform of the Barnes maze is indicated by red circle. **G** Latency to reach the target hole in each trial by the indicated mouse groups in the Barnes maze (*n* = 11 per Con, 6 per AIMP2 Tg, 7 per αSyn Tg, 9 per AIMP2;αSyn Tg mice). **H** Assessment of bradykinesia in the indicated 2.5 month old mouse groups determined by pole test (*n* = 20 per Con, 12 per AIMP2 Tg, 9 per αSyn Tg, 23 per AIMP2;αSyn Tg mice). **I** Assessment of motor coordination in the indicated 2.5 month old mouse groups determined by rotarod test (*n* = 11 per Con, 10 per AIMP2 Tg, 11 per αSyn Tg, 15 per AIMP2;αSyn Tg mice). **J** Assessment of olfactory functions of the indicated 2.5 month old mouse groups determined by hidden cookie test (*n* = 5 mice per group). Data are expressed as mean ± SEM. Statistical analysis was preformed using a one-way analysis of variance (ANOVA) test followed by Tukey’s post-hoc analysis (**B**, **E**) and Kruskal–Wallis test with Dunn’s multiple comparisons test (**C**, **G**–**J**). *p < 0.05, **p < 0.01, and ***p < 0.001.

AIMP2;αSyn mice exhibited increased total distance in the open field with modest anxiety, exceeding controls and single-Tgs (Fig. 4A–C); αSyn-only mice showed a smaller hyperlocomotor effect. Given extensive cortical/hippocampal pathology, we tested cognition. In the Y-maze, spontaneous alternation was markedly reduced in AIMP2;αSyn mice compared with controls and single-Tgs (Fig. 4D, E), indicating impaired working memory. In the Barnes maze, AIMP2;αSyn mice had increased latency across three trials, while single-Tgs performed comparably to controls by trial three (Fig. 4F, G), indicating pronounced learning/memory deficits in double-Tgs.

Motor function testing revealed bradykinesia (pole test) and impaired coordination (accelerating rotarod) specifically in AIMP2;αSyn mice (Fig. 4H, I). Consistent with olfactory bulb pathology, these mice also showed delayed hidden cookie retrieval (Fig. 4J). At a baseline age of 3 weeks—when Dox suppressed transgene expression—groups performed similarly across all tasks (Suppl. Fig. 4A–I), supporting progressive, expression-dependent behavioral decline.

### Proteomics implicates Aβ and tau-related signaling

To delineate mechanisms, we performed whole-brain (excluding cerebellum) proteomics at ~2 months post-induction. SDS-PAGE fractionation followed by MS identified 1247 shared proteins (Suppl. Fig. 5A, B; Suppl. Table 1), with 183 and 125 proteins unique to controls and AIMP2;αSyn mice, respectively. Peptide count-based quantification indicated broad expression changes (Suppl. Fig. 5C). Pathway analysis (ClueGO-Gene Ontology-Biological Processes) of proteins with > 50% change highlighted regulation of amyloid-β formation, synaptic vesicle endocytosis/recycling, translation initiation complex assembly, and amino acid metabolism (Suppl. Fig. 5D; Suppl. Table 2). Additional KEGG pathway analysis revealed significant enrichment of differentially expressed proteins in pathways related to Parkinson’s disease, prion disease, the proteasome, and endocytosis (Suppl. Fig. 5E; Suppl. Table 3). Endeavour prioritization across neurodegeneration-focused models (OMIM-seeded) ranked candidates including amyloid-β A4 protein (APP) and GSK3α (Suppl. Table 4), both central to mixed pathologies observed in LBD².

### AIMP2/αSyn co-expression drives tau signaling, soluble Aβ accumulation, and proteostatic dysfunction

**Fig. 5:**
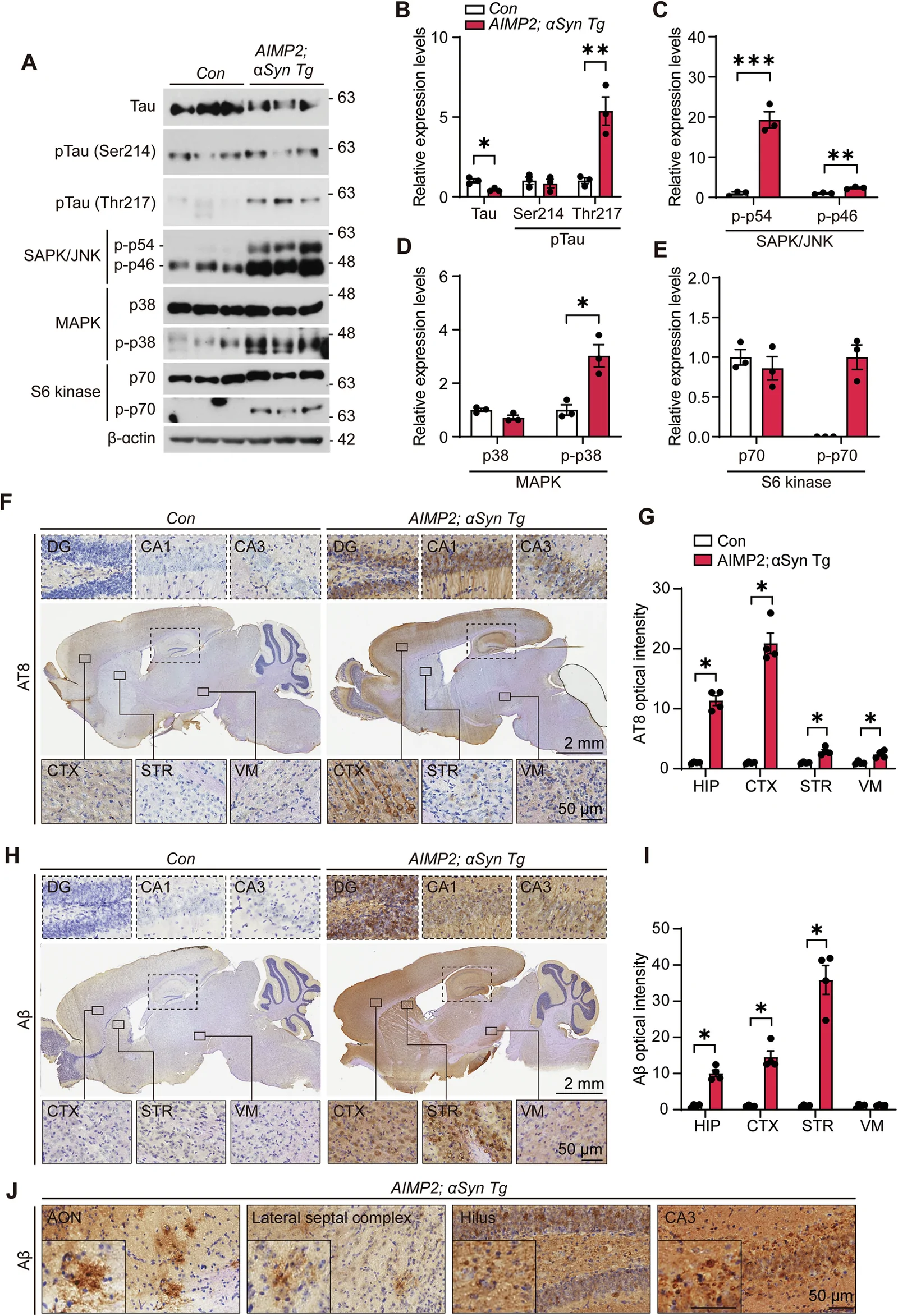
Dysregulated tau-associated signaling and development of tau and Aβ pathologies in the Lewy body dementia mouse model. **A** Representative western blots of tau, pS214-tau, pT217-tau, SAPK/JNK (p-p54, and p-p46), MAPK (p38, and p-p38), and S6 kinase (p70, and p-p70) in the whole brain lysates from 2.5 month old AIMP2;αSyn Tg and littermate control mice. β-actin serves as a loading control. **B–E** Quantification of relative expression of tau, pS214-tau, pT217-tau, SAPK/JNK (p-p54, and p-p46), MAPK (p38, and p-p38), and S6 kinase (p70, and p-p70) in the whole brains from the control and AIMP2;αSyn Tg mice normalized to β-actin (*n* = 3 mice per group). **F** Representative immunohistochemistry of pS202/pT205-Tau (AT8 clone) with Nissl counterstain in the sagittal sections of 2.5 month old AIMP2;αSyn Tg and littermate control mice. Magnified images for the selected brain subregions were also shown. Scale bar = 50 μm, and 2 mm as indicated in the images. **G** Quantification of phosphorylated Tau signals in the CTX, HIP, STR, and VM of each mouse group (*n* = 4 mice per group). **H** Representative immunohistochemistry of amyloid beta with Nissl counterstain in the sagittal sections of 2.5 month old AIMP2;αSyn Tg and littermate control mice. Magnified images for the selected brain subregions were also shown. Scale bar = 50 μm, and 2 mm as indicated in the images. **I** Quantification of amyloid beta signals in the CTX, HIP, STR, and VM of each mouse group (*n* = 4 mice per group). **J** Extracellular and intracellular distribution of amyloid beta in those indicated brain subregions of AIMP2;αSyn Tg mice monitored by anti-Aβ immunohistochemistry. Magnified images were also shown in the inset box of each image panel. Scale bar = 50 μm. AON, anterior olfactory nucleus. Data are expressed as mean ± SEM. Statistical analysis was performed using a two-tailed Student’s *t*-test (**B**–**D**) and Mann–Whitney test (E, G and I). *p < 0.05, **p < 0.01, and ***p < 0.001. The uncropped Western blot panels are provided in Suppl. fig. 10.

Given increased GSK3α peptides, we first assessed GSK3 signaling. Akt-mediated phosphorylation of GSK-3α (S21) and GSK-3β (S9) inhibits kinase activity and can prevent tau hyperphosphorylation [14, 15]. Paradoxically, AIMP2;αSyn brains showed marked increases in pS21-GSK-3α and pS9-GSK-3β versus controls (Suppl. Fig. 6A–C), with only modest increases in total GSK-3α. We screened additional tau-relevant kinases and found elevated SAPK/JNK, and MAPK activities, evidenced by increased phosphorylation of p54, p46, and p38 (Fig. 5A–D). Notably, phosphorylation of p70 S6 kinase was observed exclusively in the brains of AIMP2;αSyn Tg mice (Fig. 5A, E). Consistently, tau pT217 was increased, while pS214 was unchanged (Fig. 5A–E).

Neuropathology with AT8 antibody (pSer202/pThr205) revealed tau pathology in hippocampus and cortex, with modest signals in striatum and ventral midbrain (Fig. 5F, G). Controls and single-Tgs lacked AT8 positivity (Suppl. Fig. 6D, E). Thus, AIMP2/αSyn co-expression activates upstream kinase pathways and yields region-selective tau pathology.

Guided by the “regulation of amyloid-β formation” enrichment, we probed Aβ. AIMP2;αSyn mice displayed prominent Aβ1–42 immunoreactivity, especially in cortex, hippocampus, and striatum, whereas controls were negative (Fig. 5H, I). Signals were predominantly intracellular in affected regions, with some extracellular staining. In anterior olfactory nucleus and lateral septal complex, Aβ resembled plaque-like deposits; speckled extracellular Aβ was also observed in hippocampal hilus and CA3 (Fig. 5J). However, no changes were observed in the expression levels of amyloid precursor protein (APP) or BACE1 in AIMP2;αSyn mice compared with age-matched controls (Suppl. Fig. 7A, B). Neither AIMP2-only nor αSyn-only mice showed demonstrable Aβ accumulation (Suppl. Fig. 7C, D). Consistent with the observed Aβ immunohistochemical signals, Aβ was markedly increased in the TBS-soluble fraction of AIMP2;αSyn Tg brains but was undetectable in controls (Suppl. Fig. 7E, F), despite comparable APP levels. Aβ42 was specifically elevated in TBS-soluble lysates, with oligomeric forms detected only in Tg mice (Suppl. Fig. 7G, H). In contrast, Aβ42 in formic acid fractions and Aβ40 in all fractions were below detection limits in both groups (Suppl. Fig. 7I). Together, these data indicate that AIMP2-accelerated α-synucleinopathy drives accumulation of predominantly soluble Aβ42 species, alongside tau-associated signaling, consistent with mixed dementia pathology in LBD.

To further evaluate molecular mechanisms contributing to mixed pathologies in the LBD mouse model, lysosomal and proteasomal function were assessed by analyzing key associated proteins. In RIPA total brain lysates from 2.5-month-old LBD mice, p62 and LAMP1 levels were increased, whereas both LC3B-I and LC3B-II levels were reduced, with a modest trend of increase in the LC3B-II/I ratio compared to age-matched controls (Suppl. Fig. 8A–E). In addition, K48-linked polyubiquitinated proteins were elevated (Suppl. Fig. 8A, F). These findings indicate impaired autophagic and proteasomal degradation in LBD mouse brains.

### Progressive accumulation of mixed pathologies and reversibility upon transgene suppression in LBD mice

With continued neuronal expression of AIMP2 and αSyn, 4-month-old LBD mice exhibited further exacerbation of mixed pathologies compared to 2.5-month-old mice. Immunohistochemical analyses revealed significantly increased pS129-αSyn, AT8-positive pTau, and Aβ signals in major affected regions, including the cortex, DG, and CA1 (Suppl. Fig. 9A–G). Notably, lower brainstem regions remained largely devoid of Lewy-like pathology up to 4 months, consistent with the lack of CamKIIα-tTA–driven transgene expression in these areas. In addition, Aβ pathology showed expanded distribution, with enhanced extracellular speckled Aβ signals in CA3 and hilus regions and newly apparent deposition in the CA1 stratum lacunosum-moleculare (CA1-SLM) at 4 months (Suppl. Fig. 9H, I). Despite marked Aβ immunoreactivity, Congo red staining did not detect fibrillar amyloid plaques at 2.5 months, while only sparse, small Congo red–positive puncta emerged in the hippocampus at 4 months (Suppl. Fig. 9J).

**Fig. 6:**
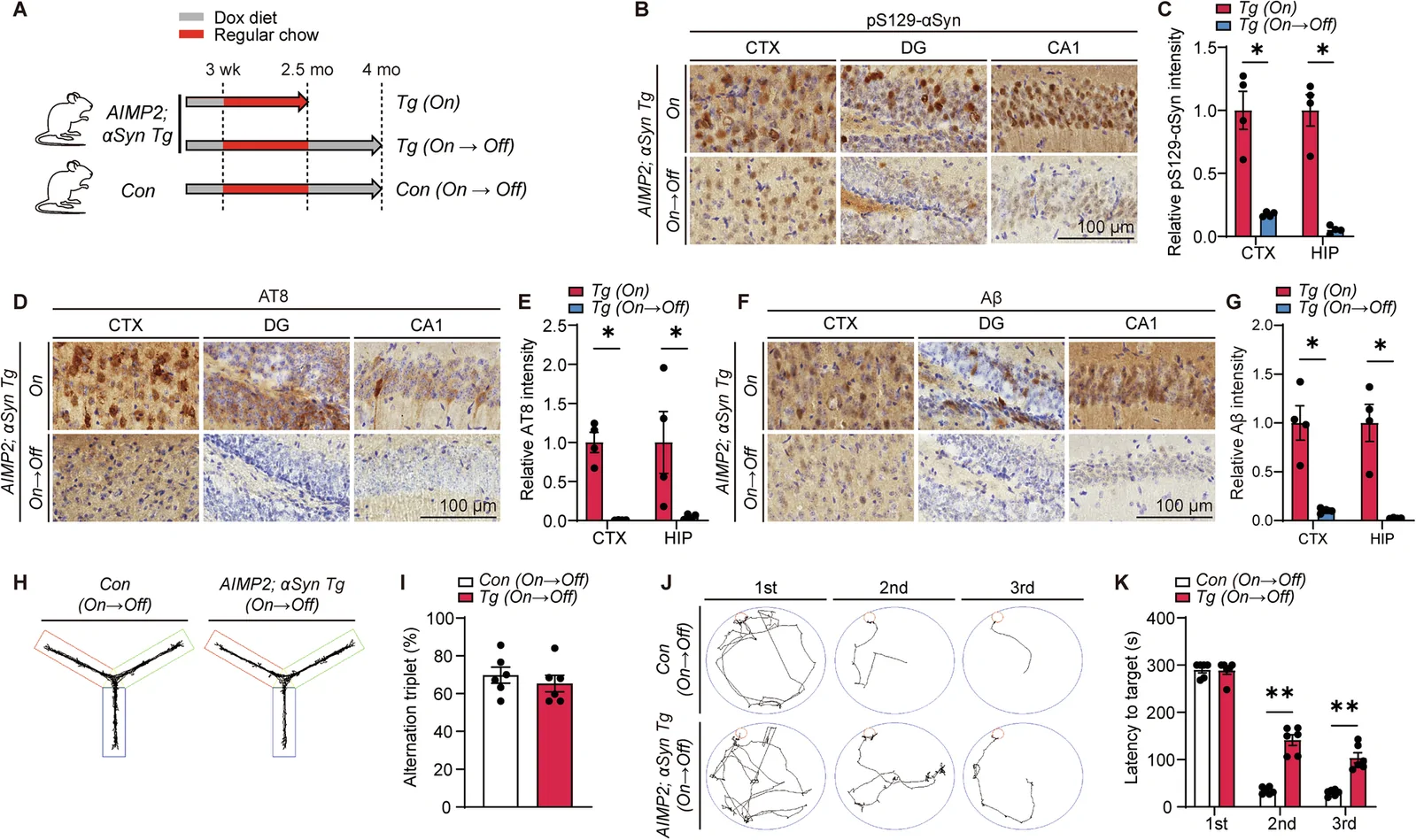
Temporal suppression of neuronal transgene expression in AIMP2;αSyn mice promotes reduction of pre-existing pathological pS129-αSyn, pTau, and Aβ, partially restoring cognitive function. **A** Schematic diagram of the experimental timeline for temporal suppression of AIMP2 and αSyn expression in conditional Tg mice. Transgene expression was induced from 3 weeks to 2.5 months (Tg (On)) and then switched off by doxycycline diet until 4 months (Tg (On→Off)). Age-matched control mice under the same regimen (Con (On→Off)) were included for baseline behavioral comparison. **B** Representative immunohistochemistry images of αSyn aggregates in the indicated brain subregions (CTX, DG, and CA1) from AIMP2;αSyn Tg mice (Tg (On) and Tg (On→Off)), detected using a pS129-αSyn–specific antibody. Sections were counterstained with Nissl. Scale bars = 100 μm. (**C**) Quantification of pS129-αSyn signals in the CTX, and HIP (DG + CA1) from AIMP2;αSyn Tg mice (Tg (On) and Tg (On→Off)) (*n* = 4 mice per group). (**D**) Representative immunohistochemistry of pS202/pT205-Tau (AT8 clone) with Nissl counterstain in the indicated brain subregions (CTX, DG, and CA1) from AIMP2;αSyn Tg mice (Tg (On) and Tg (On→Off)). Scale bar = 100 μm. **E** Quantification of AT8 signals in the CTX, and HIP (DG + CA1) from AIMP2;αSyn Tg mice (Tg (On) and Tg (On→Off)) (*n* = 4 mice per group). **F** Representative immunohistochemistry of amyloid beta with Nissl counterstain in the indicated brain subregions (CTX, DG, and CA1) from AIMP2;αSyn Tg mice (Tg (On) and Tg (On→Off)). Scale bar = 100 μm. **G** Quantification of amyloid beta signals in the CTX, and HIP (DG + CA1) of each mouse group (*n* = 4 mice per group). **H** Representative travel paths of the indicated mouse groups (4-month-old Con and Tg (On→Off)) in the Y-maze with three arms. **I** % spontaneous alteration by each mouse group in the Y-maze (*n* = 6 mice per group). **J** Representative travel paths by each mouse group in three trials (1st-trial on day 1; 2nd-trial on day 3; 3rd-trial on day 8) of Barnes maze. The location of the hole (hiding cage underneath) in the circular platform of the Barnes maze is indicated by red circle. **K** Latency to reach the target hole in each trial by the indicated mouse groups in the Barnes maze (*n* = 6 mice per group). Data are expressed as mean ± SEM. Statistical analysis was performed using the Mann–Whitney test. *p < 0.05, **p < 0.01.

To determine whether these pathologies are reversible, transgene expression was temporally suppressed by doxycycline administration from 2.5 to 4 months of age (Fig. 6A). This intervention led to a marked reduction of pre-existing pS129-αSyn, pTau, and Aβ pathologies in the cortex, DG, and CA1 (Fig. 6B–G). Functionally, this reduction in pathological protein burden was accompanied by significant improvement in cognitive performance. In the Y-maze, transgene-suppressed mice showed restoration of spontaneous alternation to levels comparable to controls (Fig. 4D, E, and Fig. 6H, I), indicating recovery of short-term working memory. In the Barnes maze, spatial learning and memory were also partially improved, as evidenced by reduced latency to locate the target hole across trials (Fig. 4F, G, and Fig. 6J, K). Specifically, the latency decreased from 209.7 s in 2.5-month-old LBD mice to 103.8 s in 4-month-old AIMP2;αSyn mice (On→Off) after 1.5 months of transgene suppression, representing a 49.5% reduction (*P* = 0.0016). Together, these findings demonstrate progressive worsening of mixed pathologies with sustained transgene AIMP2 and αSyn neuronal coexpression and their substantial reversibility upon temporal suppression, with corresponding functional recovery.

## Discussion

We describe a neuron-specific, temporally controlled AIMP2–A53T α-synuclein (αSyn) Tet-Off model that rapidly recapitulates widespread Lewy-like pathology, cortical/hippocampal degeneration with neuroinflammation, significant nigrostriatal loss, and LBD-relevant behavioral impairments. To our knowledge, this is the first genetically defined system to reproduce all three hallmark pathologies—αSyn, tau, and Aβ—without overexpressing tau or APP, while concurrently modeling cognitive, motor, and olfactory deficits within weeks. It overcomes limitations of single-Tg AIMP2 [8] or αSyn [9] mice, which do not fully capture advanced LBD/PDD phenotypes, and extends viral substantia nigra findings [5] to cortex, hippocampus, and olfactory circuits implicated in cognition and non-motor symptoms [16, 17]. Importantly, continued transgene expression further increased pS129-αSyn, pTau, and Aβ pathologies by 4 months, indicating progressive worsening of mixed proteinopathy.

Temporal control avoids developmental toxicity and enables modeling of disease onset/progression, with potential adaptability to alternative drivers or regional tTA delivery. The breadth of behavioral deficits—working/spatial memory, bradykinesia/coordination, hyperlocomotion/anxiety, and olfaction—aligned with region-specific pathology, positions this model for preclinical testing of symptomatic and disease-modifying interventions. A major advantage of this conditional system is reversibility: temporal suppression of AIMP2 and αSyn after pathology onset markedly reduced pre-existing pS129-αSyn, pTau, and Aβ accumulation in cortex and hippocampus, restored short-term working memory, and partially improved spatial memory. These findings suggest that a substantial component of the mixed pathology remains reversible at this stage, whereas incomplete behavioral rescue may reflect irreversible neuronal loss already present by 2.5 months.

A key observation is spontaneous mixed proteinopathy: Lewy-like inclusions co-occur with substantial AT8-positive tau and Aβ accumulation. Tau alterations correlated with activation of SAPK/JNK, p38, and S6K and elevated tau pT217; intriguingly, inhibitory phosphorylation of GSK-3α/β was increased, suggesting compensatory feedback or kinase crosstalk that may complicate GSK-3–targeted strategies. Region-specific tau signals (cortex/hippocampus > striatum/midbrain) mirrored Lewy-like pathology distribution, supporting a model where AIMP2-accelerated α-synucleinopathy perturbs endogenous kinase networks to promote tauopathy. Direct pathological interactions may also contribute to tau dysregulation, although this remains to be defined.

Aβ pathology in our model is evident and characterized by prominent intracellular-like Aβ immunoreactivity, with focal extracellular/plaque-like features in select regions. Early intracellular Aβ has been reported in human disease and AD models (e.g., 5XFAD) and may represent an initiating lesion preceding plaque formation [18–20]. Proteomic analyses identified alterations in pathways related to endolysosomal trafficking, proteostasis, and synaptic vesicle/translation in AIMP2;αSyn Tg brains, which may contribute to dysregulated APP processing. The biochemical data further indicate that Aβ pathology is dominated initially by TBS-soluble Aβ42, including oligomeric species, whereas Aβ42 was not detected in formic acid fractions and Aβ40 remained below detection limits. Consistent with this, Congo Red staining was negative at 2.5 months, with only sparse puncta appearing by 4 months. Thus, Aβ accumulation in this model appears to be driven mainly by soluble Aβ42 species with limited progression to fibrillar amyloid, in line with the lower aggregation propensity of rodent Aβ [21].

Unbiased proteomics supported these phenotypes, nominating pathways tied to Aβ regulation, synaptic vesicle cycling, translation initiation, and amino acid metabolism, and highlighting candidates including APP and GSK-3α. Changes in mTOR/Raptor peptides suggest mTOR pathway involvement—a regulator of autophagy and protein quality control widely implicated in neurodegeneration²⁴. This points to therapeutic strategies enhancing aggregate clearance (e.g., autophagy modulation) alongside approaches targeting αSyn seeding/spread, tau kinase signaling, and APP processing. Consistent with this, we found increased p62, LAMP1, and K48-linked polyubiquitinated proteins together with reduced LC3B-I and LC3B-II, supporting impaired lysosomal and proteasomal degradation. Because total APP and BACE1 were unchanged, these findings favor defective clearance rather than major overproduction as a contributor to Aβ accumulation, and likely also to worsening αSyn and tau pathology.

The model also has several limitations. First, because CamKIIα-tTA drives expression mainly in excitatory forebrain neurons, αSyn pathology was absent in GAD67-positive hippocampal interneurons, and inhibitory neuronal involvement is likely underrepresented. Second, although the model reproduces cognitive, motor, and olfactory deficits, hallmark LBD features such as cognitive fluctuations and visual hallucinations were not assessed. Third, the model is not optimized for Braak-like caudo-rostral propagation, as transgene expression is forebrain predominant and lower brainstem regions remained largely spared even at 4 months. Combining this model with focal αSyn PFF injection may therefore be a useful strategy to study seeded propagation in an AIMP2-high background.

Mechanistically, our data support AIMP2 as a potent accelerator of αSyn-driven pathology, although whether it is independently essential remains unresolved in this model, as doxycycline suppresses both transgenes simultaneously. The lack of demonstrable pathology in single-Tg mice and prior evidence that AIMP2 knockdown attenuates αSyn PFF-induced Lewy pathology [5] support a synergistic interaction. Future studies employing selective genetic or pharmacological targeting of AIMP2 and/or αSyn will be required to define their individual contributions across disease stages.

Although non-canonical functions of multisynthetase complex (MSC) components have been reported previously [22, 23], AIMP2 occupies a unique position because it is a well-established parkin substrate whose pathological accumulation has been extensively implicated in PD [5–7]. Thus, understanding how AIMP2 becomes available outside the MSC may provide important insight into Lewy body pathogenesis. One plausible mechanism is excessive AIMP2 accumulation. Impaired parkin-mediated degradation [6, 7], defective VPS35-dependent lysosomal clearance [24], and the proteasomal and lysosomal dysfunctions commonly observed in PD [25, 26] would increase the cytosolic pool of MSC-unbound AIMP2, facilitating its aberrant interaction with α-synuclein. Consistent with this model, our AIMP2/α-synuclein mice exhibited proteasomal and lysosomal dysfunction (Suppl. Fig. 8), suggesting a feed-forward cycle in which defective proteostasis promotes AIMP2 accumulation, whereas accumulated AIMP2 and α-synuclein further aggravate proteostasis failure. Because these degradation pathways are also responsible for clearing multiple aggregation-prone proteins, impaired proteostasis may additionally contribute to the secondary tau and Aβ pathologies observed in our model, providing a potential explanation for the mixed proteinopathy characteristic of Lewy body disorders. These findings suggest that restoring proteasomal or lysosomal function to enhance AIMP2 clearance may represent a therapeutic strategy for Lewy body disorders and mixed proteopathies.

A second, non-mutually exclusive mechanism is stress-induced mobilization of AIMP2 from the MSC through phosphorylation. Previous studies have demonstrated that DNA damage-induced JNK activation [27] and TGFβ-induced Ser156 phosphorylation [23, 28] each promote dissociation of AIMP2 from the MSC. Whether either of these mechanisms contributes to PD/LBD pathogenesis remains unknown. Our LBD model exhibited robust JNK activation (Fig. 5A, C), while oxidative stress and α-synuclein pathology are known to activate stress-responsive kinases, including c-Abl [6, 29, 30]. Together with previous reports that endogenous AIMP2 promotes α-synuclein aggregation under oxidative stress and α-synuclein PFF seeding conditions [5], these findings support a model in which PD/LBD-associated stress signaling may promote pathological MSC remodeling through AIMP2 mobilization, thereby facilitating α-synuclein pathology, proteostasis failure, and the emergence of secondary tau and Aβ pathologies. Future studies should determine whether preventing AIMP2 mobilization from the MSC can interrupt this pathogenic cascade and provide therapeutic benefit. More broadly, these findings suggest that pathological MSC remodeling may constitute an early molecular event that permits pathological interactions between AIMP2 and α-synuclein, thereby leading to proteostatic dysfunction and ultimately the mixed proteinopathy observed in LBD.

In summary, neuron-specific, inducible AIMP2–αSyn co-expression produces a rapid, reproducible, and genetically tractable LBD model that (i) captures widespread Lewy-like pathology; (ii) recapitulates cognitive, motor, and olfactory deficits; and (iii) uniquely reproduces endogenous tau and Aβ pathologies without additional transgenes. The new LBD model further shows progressive worsening with age, biochemical evidence of proteostatic dysfunction, and substantial reversibility after temporal transgene suppression. This platform enables mechanistic dissection of mixed proteinopathies and supports therapeutic discovery for LBD, PDD, and related synucleinopathies.

## Methods

### Animal experiments

All animal procedures were approved by the Institutional Animal Care and Use Committee of Sungkyunkwan University and conducted in accordance with international ethical guidelines for animal research (Approval No. SKKUIACUC2024-10-32-1). CaMKIIα-tTA (stock No. 024854) and TetP-A53T*SNCA transgenic (Tg) mice (Stock No. 012442) were obtained from The Jackson Laboratory. TetP-AIMP2 Tg mice were kindly provided by Dr. Ted M. Dawson (Johns Hopkins University). Mouse maintenance and genotyping from tail-derived genomic DNA were performed as previously described in referenced studies [8, 31]. We crossed CaMKIIα-tTA mice with TetP-A53T*SNCA;TetP-AIMP2 mice to generate single Tg (AIMP2 or α-synuclein (αSyn)) and double Tg (AIMP2;αSyn) Lewy body dementia (LBD) model mice harboring both constructs. CaMKIIα-tTA mice were used as the control group (Con), and CaMKIIα-tTA;TetP-AIMP2 (AIMP2 Tg), CaMKIIα-tTA;TetP-A53T*SNCA (αSyn Tg), and CaMKIIα-tTA;TetP-AIMP2;TetP-A53T*SNCA (AIMP2;αSyn Tg) mice were designated as the experimental groups. Mice were backcrossed to a C57BL/6 background, and both male and female littermates were used in all experiments. All animals used in this study were included in subsequent analyses. The number of mice used for each experiment was determined with reference to previous studies in the same research field and using similar technical approaches. No randomization was applied for colony maintenance or group allocation. Experimenters conducting behavioral and neuropathological analyses were blinded to the experimental group of each mouse. Mice were maintained on a doxycycline-containing diet (200 mg/kg diet, DooYeolBiotech) ad libitum until 3 weeks of age to suppress transgene expression during development. Suppression of AIMP2 and αSyn transgene expression was confirmed by Western blot analysis of brain lysates (Suppl. Fig. 1B). Transgene expression was subsequently induced by switching to regular chow and maintained until 2.5 months of age. For temporal suppression of transgene expression after disease onset, 2.5-month-old LBD mice were switched back to the doxycycline-containing diet and maintained for an additional 1.5 months until 4 months of age.

### Protein preparation and western Blot

Whole brains from control and AIMP2;αSyn mice were powdered on dry ice and sequentially fractionated. For total lysates, tissue was homogenized in lysis buffer (10 mM Tris–HCl, 150 mM NaCl, 5 mM EDTA, 0.5% NP-40, 0.5% sodium deoxycholate; pH 7.4) with protease/phosphatase inhibitors, followed by centrifugation at 14,000 × g for 30 min at 4 °C. For soluble and insoluble fractions, homogenization was performed using NP-40 buffer for the soluble fraction, and SDS-containing buffer (RIPA buffer) for the insoluble fraction, with identical centrifugation conditions. Additionally, to preserve mildly soluble proteins, tissues were homogenized in TBS-soluble buffer (10 mM Tris–HCl, 150 mM NaCl) with protease/phosphatase inhibitors. followed by centrifugation under identical conditions. Protein concentration was determined by BCA assay (Pierce). Samples were mixed with Laemmli buffer, separated by SDS–PAGE, and transferred to nitrocellulose membranes. Immunoblotting was performed using primary antibodies and chemiluminescent detection. Band intensity was quantified using ImageJ.

### Immunofluorescence

Fixed mouse brain sections were incubated in blocking buffer (0.1% Triton X-100, 5% goat serum in PBS) for 1 h at room temperature, followed by overnight incubation with primary antibodies at 4 °C. Sections were washed, incubated with fluorescent secondary antibodies for 1 h at room temperature, and counterstained with DAPI. Images were acquired by fluorescence and confocal microscopy (Zeiss LSM710) and analyzed using ZEN and Imaris software.

### Immunohistochemistry

Mice were perfused with PBS and 4% paraformaldehyde. Brains were post-fixed overnight, cryoprotected in 30% sucrose, sectioned, and incubated in blocking buffer. Sections were incubated with primary antibodies overnight at 4 °C, followed by biotinylated secondary antibodies and visualization with DAB. Nissl staining was used for counterstaining.

### Transmission electron microscopy

Hippocampal tissue was fixed in 2.5% glutaraldehyde and 0.1 M cacodylate buffer, post-fixed in osmium tetroxide/potassium ferrocyanide, dehydrated, and embedded in Epon 812. Ultrathin sections (60 nm) were cut, mounted on copper grids, and stained with uranyl acetate and lead citrate. Synapses were imaged at 120 kV (JEOL JEM-1400) and quantified using ImageJ.

### Statistics

Data are presented as mean ± SEM with individual data points shown where feasible. Normality of residuals was assessed using the Shapiro–Wilk test. Homogeneity of variances was tested using the F test for two-group comparisons and the Brown–Forsythe test for ≥ 3 groups. For two-group comparisons, unpaired two-tailed t-tests were used when assumptions were met; otherwise, the Mann–Whitney U test was applied. For ≥ 3 groups, one-way ANOVA with Tukey’s post hoc test was used when assumptions were met; otherwise, the Kruskal–Wallis test with Dunn’s multiple comparisons test was applied. Exact n (biological replicates) is reported in figure legends. *P* < 0.05 was considered statistically significant. All analyses were conducted using GraphPad Prism (v8.4.2).

## Supplementary information

Supplementary information

Full blots

Supplementary table 1

Supplementary table 2

Supplementary table 3

Supplementary table 4

## Data Availability

Original data for this manuscript are available on reasonable request. The TEM raw data was deposited in K-BDS (Korea BioData Station, https://kbds.re.kr) with the accession ID KAP241598. https://kbds.re.kr/reviewer/9e7d6356-903a-4f6e-ada2-862518919006-KAP241598?lang=en.

## References

[1] Sanford AM. Lewy body dementia. Clin Geriatr Med. 2018;34:603–15. 10.1016/j.cger.2018.06.007 30336990

[2] Robinson JL, Lee EB, Xie SX, Rennert L, Suh E, Bredenberg C, et al. Neurodegenerative disease concomitant proteinopathies are prevalent, age-related and APOE4-associated. Brain. 2018;141:2181–93. 10.1093/brain/awy146 PMC6022546 29878075

[3] Lee MK, Stirling W, Xu Y, Xu X, Qui D, Mandir AS, et al. Human alpha-synuclein-harboring familial Parkinson’s disease-linked Ala-53 -> Thr mutation causes neurodegenerative disease with alpha-synuclein aggregation in transgenic mice. Proc Natl Acad Sci USA. 2002;99:8968–73. 10.1073/pnas.132197599 PMC124407 12084935

[4] Kim S, Kwon SH, Kam TI, Panicker N, Karuppagounder SS, Lee S, et al. Transneuronal Propagation of Pathologic alpha-Synuclein from the Gut to the Brain Models Parkinson’s Disease. Neuron. 2019;103:627–641 e627. 10.1016/j.neuron.2019.05.035 PMC6706297 31255487

[5] Ham S, Yun SP, Kim H, Kim D, Seo BA, Kim H. Amyloid-like oligomerization of AIMP2 contributes to alpha-synuclein interaction and Lewy-like inclusion. Sci Transl Med. 2020;12:eaax0091 10.1126/scitranslmed.aax0091 PMC8082540 33177178

[6] Ko HS, Lee Y, Shin JH, Karuppagounder SS, Gadad BS, Koleske AJ, et al. Phosphorylation by the c-Abl protein tyrosine kinase inhibits parkin’s ubiquitination and protective function. Proc Natl Acad Sci USA. 2010;107:16691–6. 10.1073/pnas.1006083107 PMC2944759 20823226

[7] Ko HS, von Coelln R, Sriram SR, Kim SW, Chung KK, Pletnikova O, et al. Accumulation of the authentic parkin substrate aminoacyl-tRNA synthetase cofactor, p38/JTV-1, leads to catecholaminergic cell death. J Neurosci. 2005;25:7968–78. 10.1523/JNEUROSCI.2172-05.2005 PMC6725452 16135753

[8] Lee Y, Karuppagounder SS, Shin JH, Lee YI, Ko HS, Swing D, et al. Parthanatos mediates AIMP2-activated age-dependent dopaminergic neuronal loss. Nat Neurosci. 2013;16:1392–1400. 10.1038/nn.3500 PMC3785563 23974709

[9] Nuber S, Petrasch-Parwez E, Winner B, Winkler J, von Horsten S, Schmidt T, et al. Neurodegeneration and motor dysfunction in a conditional model of Parkinson’s disease. J Neurosci. 2008;28:2471–84. 10.1523/JNEUROSCI.3040-07.2008 PMC6671187 18322092

[10] Lim Y, Kehm VM, Li C, Trojanowski JQ, Lee VM. Forebrain overexpression of alpha-synuclein leads to early postnatal hippocampal neuron loss and synaptic disruption. Exp Neurol. 2010;221:86–97. 10.1016/j.expneurol.2009.10.005 PMC2812632 19833127

[11] Jo A, Lee Y, Kam TI, Kang SU, Neifert S, Karuppagounder SS. PARIS farnesylation prevents neurodegeneration in models of Parkinson’s disease. Sci Transl Med. 2021;13:eaax8891 10.1126/scitranslmed.aax8891 PMC9990146 34321320

[12] Mayford M, Bach ME, Huang YY, Wang L, Hawkins RD, Kandel ER. Control of memory formation through regulated expression of a CaMKII transgene. Science. 1996;274:1678–83. 10.1126/science.274.5293.1678 8939850

[13] Yetman MJ, Jankowsky JL. Wild-type neural progenitors divide and differentiate normally in an amyloid-rich environment. J Neurosci. 2013;33:17335–41. 10.1523/JNEUROSCI.1917-13.2013 PMC3812504 24174666

[14] Cross DA, Alessi DR, Cohen P, Andjelkovich M, Hemmings BA. Inhibition of glycogen synthase kinase-3 by insulin mediated by protein kinase B. Nature. 1995;378:785–9. 10.1038/378785a0 8524413

[15] Srivastava AK, Pandey SK. Potential mechanism(s) involved in the regulation of glycogen synthesis by insulin. Mol Cell Biochem. 1998;182:135–41. 9609122

[16] Adler CH, Beach TG. Neuropathological basis of nonmotor manifestations of Parkinson’s disease. Mov Disord. 2016;31:1114–9. 10.1002/mds.26605 PMC4981515 27030013

[17] Koeglsperger T, Rumpf SL, Schliesser P, Struebing FL, Brendel M, Levin J, et al. Neuropathology of incidental Lewy body & prodromal Parkinson’s disease. Mol Neurodegener. 2023;18:32. 10.1186/s13024-023-00622-7 PMC10182593 37173733

[18] Gouras GK, Almeida CG, Takahashi RH. Intraneuronal Abeta accumulation and origin of plaques in Alzheimer’s disease. Neurobiol Aging. 2005;26:1235–44. 10.1016/j.neurobiolaging.2005.05.022 16023263

[19] Gouras GK, Tsai J, Naslund J, Vincent B, Edgar M, Checler F, et al. Intraneuronal Abeta42 accumulation in human brain. Am J Pathol. 2000;156:15–20. 10.1016/s0002-9440(10)64700-1 PMC1868613 10623648

[20] Oakley H, Cole SL, Logan S, Maus E, Shao P, Craft J, et al. Intraneuronal beta-amyloid aggregates, neurodegeneration, and neuron loss in transgenic mice with five familial Alzheimer’s disease mutations: potential factors in amyloid plaque formation. J Neurosci. 2006;26:10129–40. 10.1523/JNEUROSCI.1202-06.2006 PMC6674618 17021169

[21] Jankowsky JL, Younkin LH, Gonzales V, Fadale DJ, Slunt HH, Lester HA, et al. Rodent A beta modulates the solubility and distribution of amyloid deposits in transgenic mice. J Biol Chem. 2007;282:22707–20. 10.1074/jbc.M611050200 PMC4435736 17556372

[22] Wang Q, Yu H, Yuan X, Li R, Li X, Yin S, et al. Dopaminergic Neuron-Derived AIMP1 promotes neurodegeneration via CD23-dependent microglial activation. CNS Neurosci Ther. 2025;31:e70472. 10.1111/cns.70472 PMC12168485 40522094

[23] Zhou Z, Sun B, Huang S, Yu D, Zhang X. Roles of aminoacyl-tRNA synthetase-interacting multi-functional proteins in physiology and cancer. Cell Death Dis. 2020;11:579. 10.1038/s41419-020-02794-2 PMC7382500 32709848

[24] Yun SP, Kim H, Ham S, Kwon SH, Lee GH, Shin JH, et al. VPS35 regulates parkin substrate AIMP2 toxicity by facilitating lysosomal clearance of AIMP2. Cell Death Dis. 2017;8:e2741. 10.1038/cddis.2017.157 PMC5477581 28383562

[25] Dehay B, Martinez-Vicente M, Caldwell GA, Caldwell KA, Yue Z, Cookson MR, et al. Lysosomal impairment in Parkinson’s disease. Mov Disord. 2013;28:725–32. 10.1002/mds.25462 PMC5131721 23580333

[26] McNaught KS, Jackson T, JnoBaptiste R, Kapustin A, Olanow CW. Proteasomal dysfunction in sporadic Parkinson’s disease. Neurology. 2006;66:S37–49. 10.1212/wnl.66.10_suppl_4.s37 16717251

[27] Han JM, Park BJ, Park SG, Oh YS, Choi SJ, Lee SW, et al. AIMP2/p38, the scaffold for the multi-tRNA synthetase complex, responds to genotoxic stresses via p53. Proc Natl Acad Sci USA. 2008;105:11206–11. 10.1073/pnas.0800297105 PMC2516205 18695251

[28] Kim DG, Lee JY, Lee JH, Cho HY, Kang BS, Jang SY, et al. Oncogenic Mutation of AIMP2/p38 Inhibits Its Tumor-Suppressive Interaction with Smurf2. Cancer Res. 2016;76:3422–36. 10.1158/0008-5472.CAN-15-3255 27197155

[29] Karuppagounder SS, Wang H, Kelly T, Rush R, Nguyen R, Bisen S, et al. The c-Abl inhibitor IkT-148009 suppresses neurodegeneration in mouse models of heritable and sporadic Parkinson’s disease. Sci Transl Med. 2023;15:eabp9352. 10.1126/scitranslmed.abp9352 PMC13248603 36652533

[30] Kim JH, Yang S, Kim H, Vo DK, Maeng HJ, Jo A, et al. Preclinical studies and transcriptome analysis in a model of Parkinson’s disease with dopaminergic ZNF746 expression. Mol Neurodegener. 2025;20:24. 10.1186/s13024-025-00814-3 PMC11871723 40022229

[31] Lin X, Parisiadou L, Gu XL, Wang L, Shim H, Sun L, et al. Leucine-rich repeat kinase 2 regulates the progression of neuropathology induced by Parkinson’s-disease-related mutant alpha-synuclein. Neuron. 2009;64:807–27. 10.1016/j.neuron.2009.11.006 PMC2807409 20064389

