# Supplementary material for: Neuronal AIMP2–α-synuclein synergy drives endogenous tau and amyloid-β pathologies and neurodegeneration in a mechanistic model of Lewy body dementia
Source: Cell Death Dis. 2026 Aug 13;17(1):959. doi: 10.1038/s41419-026-09181-3 (PMC13752285; doi:10.1038/s41419-026-09181-3)
Supplement: Supplementary file 1 [file 41419_2026_9181_MOESM1_ESM.pdf]

Supplementary information

Neuronal AIMP2–α-synuclein synergy drives endogenous tau and amyloid-β pathologies and neurodegeneration in a mechanistic model of Lewy body dementia

Doeun Kim, Heejeong Kim, Ji Hun Kim, Hyojung Kim, Joo-Ho Shin, Han Seok Ko, Eunyoung Moon, Hee-Seok Kweon, Yunjong Lee

Supplementary methods

Chemicals and antibodies

The following primary antibodies were used: rabbit antibody to JTV-1 (Proteintech, #10424-1-AP), mouse antibody to α-synuclein (αSyn) (BD, #610787), mouse antibody to phospho-S129-αSyn (Wako, #015-25191) (BioLegend, #825701), rabbit antibody to GFAP (Abcam, #ab7260), rabbit antibody to Iba1 (Wako, #019-19741), rabbit antibody to tyrosine hydroxylase (Novus Biologicals, #NB300-109), mouse antibody to tyrosine hydroxylase (ImmunoStar, #22941), rabbit antibody to Tau (Cell Signaling Technology, #46687), rabbit antibody to phospho-Tau (Ser214) (Cell Signaling Technology, #77348, rabbit antibody to phosphor-Tau (Thr217) (Cell Signlaing Technology #51625), rabbit antibody Phospho-SAPK/JNK (Thr183/Tyr185) (Cell Signaling Technology, # 4668), rabbit antibody to p38 MAPK (Cell Signaling Technology, #8690), rabbit antibody to phospho-p38 MAPK (Thr180/Tyr182) (Cell Signaling Technology, #4511), rabbit antibody to p70 S6 Kinase (Cell Signaling Technology, # 34475), rabbit antibody to phospho-p70 S6 Kinase (Thr389) (Cell Signaling Technology, # 97596), rabbit antibody to GSK3α (Cell Signaling Technology, #4337), rabbit antibody to phospho-Ser21-GSK3α (Cell Signaling Technology, #9316), rabbit antibody to GSK3β (Cell Signaling Technology, #12456), rabbit antibody to phospho-Ser9-GSK3β (Cell Signaling Technology, #5558), rabbit antibody to Phospho-GSK-3α/β (Ser21/9) (Cell Signaling Technology # 8566), mouse antibody to Phospho-Tau (Ser202, Thr205) Monoclonal Antibody (AT8) (Invitrogen, #MN1020), rabbit antibody to beta Amyloid (Invitrogen, # 700254), mouse antibody to APP/Aβ (Cell Signaling Technology, #2450), rabbit antibody to BACE1 (Cell Signaling Technology, #5606), rabbit antibody to Aβ40 (Invitrogen, #44-136), rabbit antibody to p62 (Cell Signaling Technology, #5114), rabbit antibody to LC3B (Invitrogen, #PA1-16930), rabbit antibody to Lamp1 (BD, #555798), rabbit antibody to K48-pUb (Cell Signaling Technology, #8081).

The following secondary antibodies were used : HRP-conjugated goat antibody to rabbit IgG (Genetex, #Gtx-213110-01), HRP-conjugated goat antibody to mouse IgG (Genetex, #Gtx-213111-01), biotin-conjugated goat antibody to mouse IgG (Vector Laboratories, #BA-9200), biotin-conjugated goat antibody to rabbit IgG (Vector Laboratories, #BA-1000), HRP-conjugated mouse antibody β-Actin (Sigma–Aldrich, #A3854), Alexa Fluor 488 donkey antibody mouse IgG (Invitrogen, #A21202), Alexa Fluor 488 goat antibody rabbit IgG (Invitrogen, #A11008), Alexa Fluor 568 donkey antibody mouse IgG (Invitrogen, #A10037), Alexa Fluor 568 donkey

antibody rabbit IgG (Invitrogen, #A10042).

TH stereology and Nissl counting

Stereological analysis was performed to estimate the total number of TH-positive neurons in the substantia nigra pars compacta (SNpc), visualized by DAB staining. Cell counting was conducted using the optical fractionator method implemented in Stereo Investigator software (MicroBrightField, Williston, VT). Similar stereological quantification was applied to Nissl-stained neurons in the cortex, and hippocampal subregions using coronal brain sections. All stereological assessments were carried out by an experimenter blinded to the experimental mouse groups.

Behavior tests

Open field test

Locomotion and exploration were assessed in open field using a square chamber (40 × 40 × 40 cm) divided into 64 (8 × 8) equal area. The central area contained 4 central compartments (2 × 2). The peripheral area contained 12 compartments surrounding the center region, and the edge area made up the rest of squares. Each mouse was placed in a dark closed box in the center and left for 15miniutes. Each trial utilized Smart v3.0 software for monitoring the distance covered, duration within the center, and entries made into the center, serving as indicators of anxiolytic activity.

Pole test

The pole is a bandaged rod that is 58 cm long and 10mm in diameter. For pole test, the mice adapted the cage to be tested for at least 5minutes. Then, the mice were placed at the top of the stick facing down. The total time was recorded until it reached the floor. The latency calculated the average time of the 2 trails.

Rotarod test

Before the test trial, mice were trained to walk in the rotarod for 2days. On the test day (day 3), the mice were placed on the accelerating cylinder and recorded the latency and speed to fell off the rotarod. The rotarod speed was incrementally raised from 4 rpm to 40 rpm within 5 min. The measurement was recorded when the mouse fell from the rotarod.

Hidden cookie finding test

The mice were familiar with the cookie for a week before the test. The cookie (~2.5 g) was placed into the cage atop the bedding every 48 hours, repeated three times. Subsequently, the mice underwent a 3-day deprivation period prior to testing. At the following day, mice were individually caged with 1 cm of fresh bedding. Initially, a piece of cookie was concealed in one corner of the cage and the time taken to locate it was recorded. An hour later, another cookie was placed in the same site as the first trial, and the behavior was observed and analyzed. The next day, the piece of cookie was hidden in a different corner than the previous day, and this process was repeated twice, following the same protocol as the first day of the experiment.

Y maze

Y maze was performed to examine the cognition and memory levels. The Y-maze task consisted of three arms (red, yellow, and blue) were 120° apart, 35 cm long, 10 cm wide, and 15cm high. The mice were placed in the maze for 300 sec, and tracked by Smart v3.0 software to record the traveled distance and to count the arm visit triplets as an indicator of locomotor activity.

Barnes maze

The Barnes maze task used to examine hippocampal-dependent spatial learning and memory. The maze is a 90 cm diameter round acrylic plate with 20 holes 5 cm in diameter, and one of them is only the hole has escape space (target) for the mouse to hide. Mouse traces were monitored with an overhead video camera controlled by Smart v3.0 software. 3days of training trials were performed with a maximum of 300 seconds, and the escape box location was changed for every trail. During each trail, the mouse's escape latency was recorded from the center of the round plate to the escape hole. The probe trial day was conducted at intervals of 1, 3, and 5days, and the location of the escape box in each trail was fixed. The escape latency and escape velocity were quantified after each test. The escape latency and escape velocity were quantified afterward using Smart v3.0

software.

Mass spectrometry and proteomic analysis

Whole-brain lysates (excluding cerebellum) from control and LBD model mice were subjected to mass spectrometry-based proteomic analysis. Proteins were separated by SDS-PAGE, excised, digested with trypsin, and analyzed using an ESI-Orbitrap mass spectrometer (Q Exactive Plus, Thermo Fisher Scientific) operated in data-dependent acquisition mode. The top 10 most intense ions from each full MS scan were selected for MS/MS fragmentation. The spray voltage was set to 2.1 kV, and the ion transfer tube temperature was maintained at 150°C. Samples were introduced via nano-flow high-performance liquid chromatography (nanoHPLC) using an Ultimate 3000 RSLCnano system (Thermo Fisher). Peptides were first loaded onto a trap column (Acclaim PepMap C18, 5 µm, 100 Å, 2 cm × 100 µm) and separated on an analytical column (Easy-Spray C18, 3 µm, 100 Å, 15 cm × 75 µm) at a flow rate of 300 nL/min. Raw data files were converted to Mascot generic format (MGF) using tools provided by Thermo Fisher. Database searching was performed using Mascot version 2.5 against the UniProt mouse protein database. Up to two missed tryptic cleavages were allowed. Protein identifications and relative quantification were performed using Scaffold software (Proteome Software Inc.) following Mascot analysis. Differentially expressed proteins were identified based on total spectral counts, and group comparisons were normalized using a minimum value of 1.0

Cytoscape and Endeavour bioinformatic analysis

Differentially expressed proteins (DEPs) were defined based on ≥ 50% change in peptide counts between LBD mouse brains and controls. Functional enrichment analysis was performed using Cytoscape (v3.10.1) with the ClueGO plugin (v2.5.x), including Gene Ontology (Biological Process) and KEGG pathway databases for *Mus musculus* (Gene Symbol identifiers, all evidence codes). Enrichment was assessed using a two-sided hypergeometric test with Bonferroni step-down correction. For GO analysis, a significance cutoff of $p < 0.01$ was applied with GO levels restricted to 3–8. For KEGG analysis, a significance cutoff of $p < 0.05$ was used with a minimum gene percentage of 5 %. Functional grouping was performed using a kappa score threshold of

0.4, minimum group size of 1, and ≥ 50 % gene overlap for merging. From 474 input IDs (465–470 recognized genes), 453 genes (97.4%) were annotated in GO and 268 genes (57.0%) in KEGG. After significance filtering, 54 GO terms (138 genes) and 9 KEGG pathways (89 genes) were retained for downstream interpretation. Proteins with differential peptide counts between LBD mouse brains and controls were also prioritized for relevance to Parkinson's disease and other neurodegenerative disorders using the Endeavour algorithm[2]. A training gene set was created by querying 348 genes from the OMIM database using the search terms "neurodegenerative disease," "Parkinson," "Alzheimer," and "Huntington." Model generation in Endeavour incorporated multiple data sources, including: Gene Ontology annotations, InterPro and Pfam domains, text mining, Reactome pathways, RGD pathways, and protein–protein interaction databases (BioGRID and STRING). LBD candidate genes were identified by reanalyzing proteins with altered peptide counts in whole-brain lysates from LBD versus control mice. All differentially detected proteins were evaluated against the trained models, and individual rankings were generated for each data source. The top 30 candidate genes were determined based on the integrated global ranking across all models.

Congo Red staining

Fixed mouse brain sections were washed with PBS and stained with alkaline congo red solution (0.1 % Congo red; Sigma-Aldrich, #C6277 in 80% ethanol containing 0.1% NaOH and saturated NaCl) for 30 min at RT. Sections were then differentiated in 80% ethanol for 15 sec, followed by washing in PBS. Sections were mounted using immuno mounting medium.

Supplementary figures

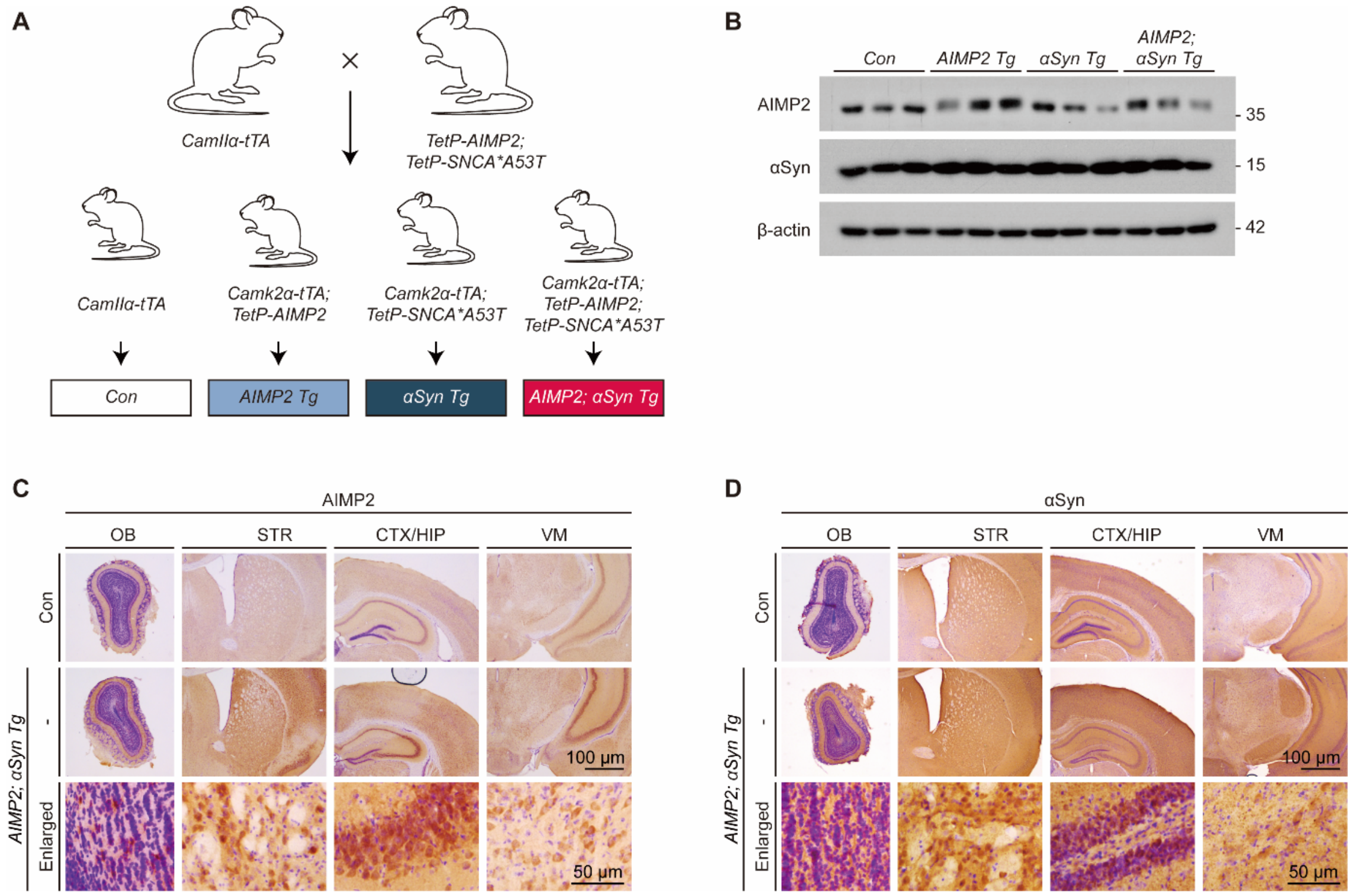

Supplementary figure 1. Tet-Off conditional expression of AIMP2 and α-synuclein (αSyn) (A53T) in various brain regions of AIMP2;αSyn transgenic (Tg) mouse

(A) Breeding scheme of CamKIIα-tTA driver mice and TetP-AIMP2;TetP-αSyn-A53T double Tg responder mice to generate triple Tg mice (designated as AIMP2; αSyn Tg) that express both AIMP2 and A53T-αSyn in brains by pan-neuronally active CamKIIα promoter induced tTA. CamKIIα-tTA was used as littermate control. CamKIIα-tTA;TetP-AIMP2 and CamKIIα-tTA;TetP-SNCA-A53T were designated as AIMP2 Tg and αSyn Tg, respectively. CamKIIα-tTA was used as a control.

(B) Suppression of AIMP2 and αSyn expression in the whole brains from the each three-week old mouse group that were fed with doxycycline diet since the mating of their parents mice determined by western blot using AIMP2 and αSyn specific antibodies.

(C) Representative anti-AIMP2 immunohistochemistry images of olfactory bulb (OB), striatum (STR),

cortex/hippocampus (CTX/HIP), and ventral midbrains (VM) coronal sections from 2.5 month old AIMP; αSyn Tg mice and littermate control. Enlarged images of the Tg mouse brain sections are shown in the bottom panel. Scale bar = 100, 50 μm, respectively.

(D) Representative anti-αSyn immunohistochemistry images of olfactory bulb (OB), striatum (STR), cortex/hippocampus (CTX/HIP), and ventral midbrains (VM) coronal sections from 2.5 month old AIMP; αSyn Tg mice and littermate control. Enlarged images of the Tg mouse brain sections are shown in the bottom panel. Scale bar = 100, 50 μm, respectively. The uncropped Western blot panels are provided in Supplementary figure 10.

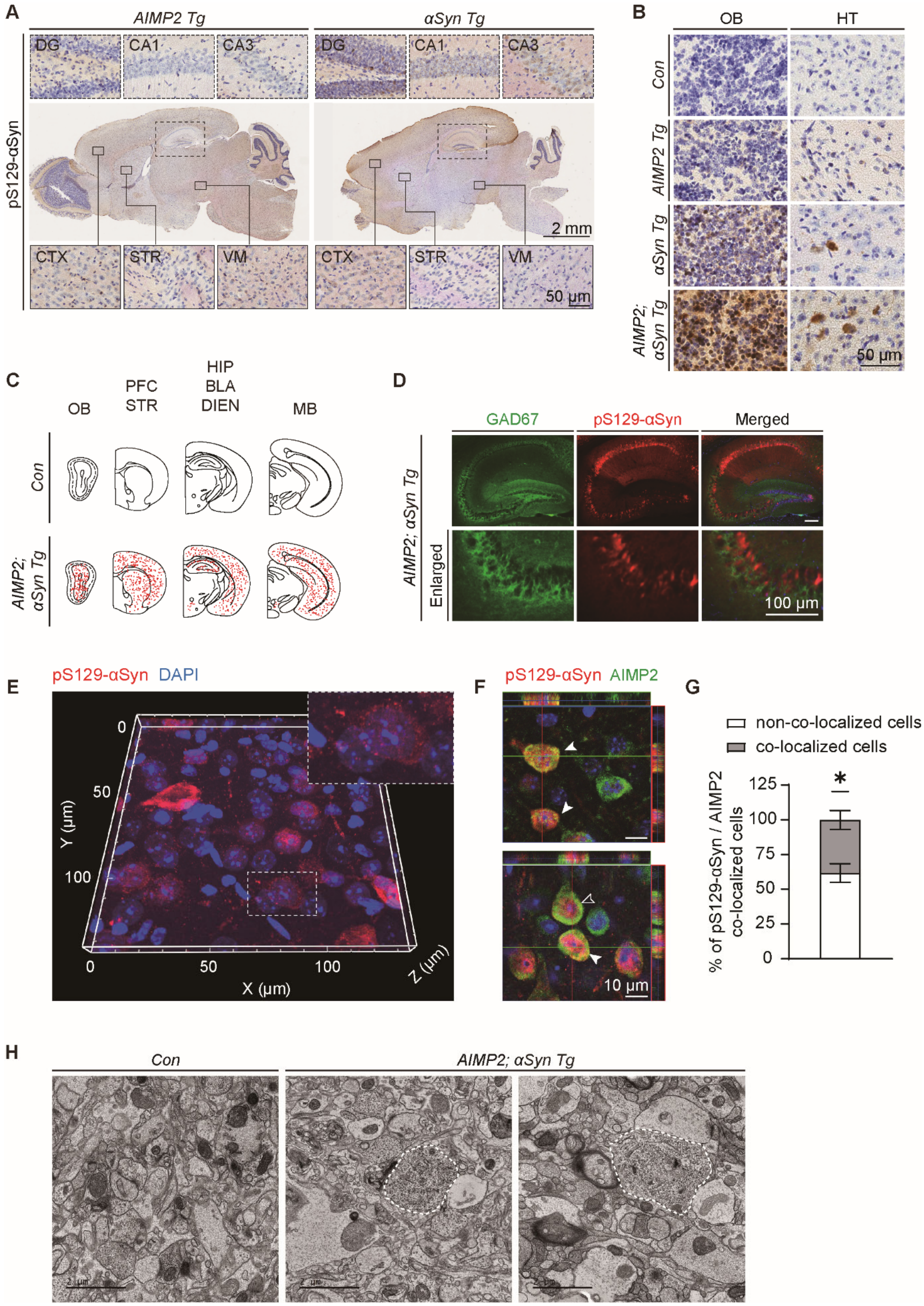

A
AIMP2 Tg
αSyn Tg
pS129-αSyn
DG
CA1
CA3
CTX
STR
VM
2 mm
50 µm
B
OB
HT
Con
AIMP2 Tg
αSyn Tg
AIMP2; αSyn Tg
50 µm
C
OB
PFC STR
HIP BLA DIEN
MB
Con
AIMP2; αSyn Tg
D
GAD67
pS129-αSyn
Merged
AIMP2; αSyn Tg
Enlarged
100 µm
E
pS129-αSyn DAPI
Y (µm)
X (µm)
Z (µm)
0
50
100
F
pS129-αSyn AIMP2
10 µm
G
non-co-localized cells
co-localized cells
% of pS129-αSyn / AIMP2 co-localized cells
125
100
75
50
25
0
*
H
Con
AIMP2; αSyn Tg

1

Supplementary figure 2. Neuronal expression of both AIMP2 and αSyn led to wide spread αSyn aggregate pathologies.

(A) Representative immunohistochemistry images showing no demonstrable Lewy-like inclusions in the sagittal sections from 2.5 month old mouse group of the indicated genotypes monitored by using pS129-αSyn specific antibody. Enlarged images were also shown. Scale bar = 2 mm for regular view and 50 um for enlarged images.

(B) Representative immunohistochemistry images showing Lewy-like inclusions in the olfactory bulb (OB), hypothalamus (HT) sections from the indicated genotypes monitored by using pS129-αSyn specific antibody. Scale bar = 50 μm.

(C) Quantitative dot labeling of pS129-αSyn expression in each brain regions of 2.5 month old AIMP2;αSyn Tg and littermate control integrated by anti-pS129-αSyn immunohistochemistry image analysis.

(D) Representative immunofluorescence images of pS129-αSyn and GAD67 (an inhibitory neuron marker) in the hippocampus (HIP) of 2.5 month old AIMP2;αSyn Tg mice. Higher-magnification images are shown in the bottom panels. Scale bar = 100 μm.

(E) 3D reconstructed immunofluorescence images showing distribution of pS129-αSyn labeled Lewy-like inclusion in the cortex of AIMP2;αSyn Tg mice. An enlarged inset highlights the perinuclear presence of pS129-αSyn puncta is also included. Nuclei were counterstained with DAPI.

(F) Confocal z-stack imaging of AIMP2 (green signal) and pS129-αSyn (red signal) in a neuron of AIMP2;αSyn Tg mice with z-stack. Filled arrow heads indicate neurons with overlapping AIMP2 and pS129-αSyn signals, while empty arrow heads indicate neurons without obvious colocalization of AIMP2 and pS129-αSyn immunofluorescent signals. Scale bar = 10 μm.

(G) Percentage of neurons showing overlap between pS129-αSyn and AIMP2 signals among cells expressing both in the cortex of the AIMP2;αSyn Tg mice (n = 81 cells from 4 mice). Statistical analysis was performed using a two-tailed Student's t-test. *$p < 0.05$, **$p < 0.01$, and ***$p < 0.001$.

(H) Ultrastructural images of cortex sections from 2.5-month-old AIMP2;αSyn Tg mice and littermate control.

AIMP2;αSyn Tg mice, compared to controls, exhibited Lewy-like inclusions (delineated with dotted lines) containing fibrillar aggregates and fragments of membranous structures derived from disrupted mitochondria, numerous autophagic vacuoles, and electron-dense lipofuscin granules.

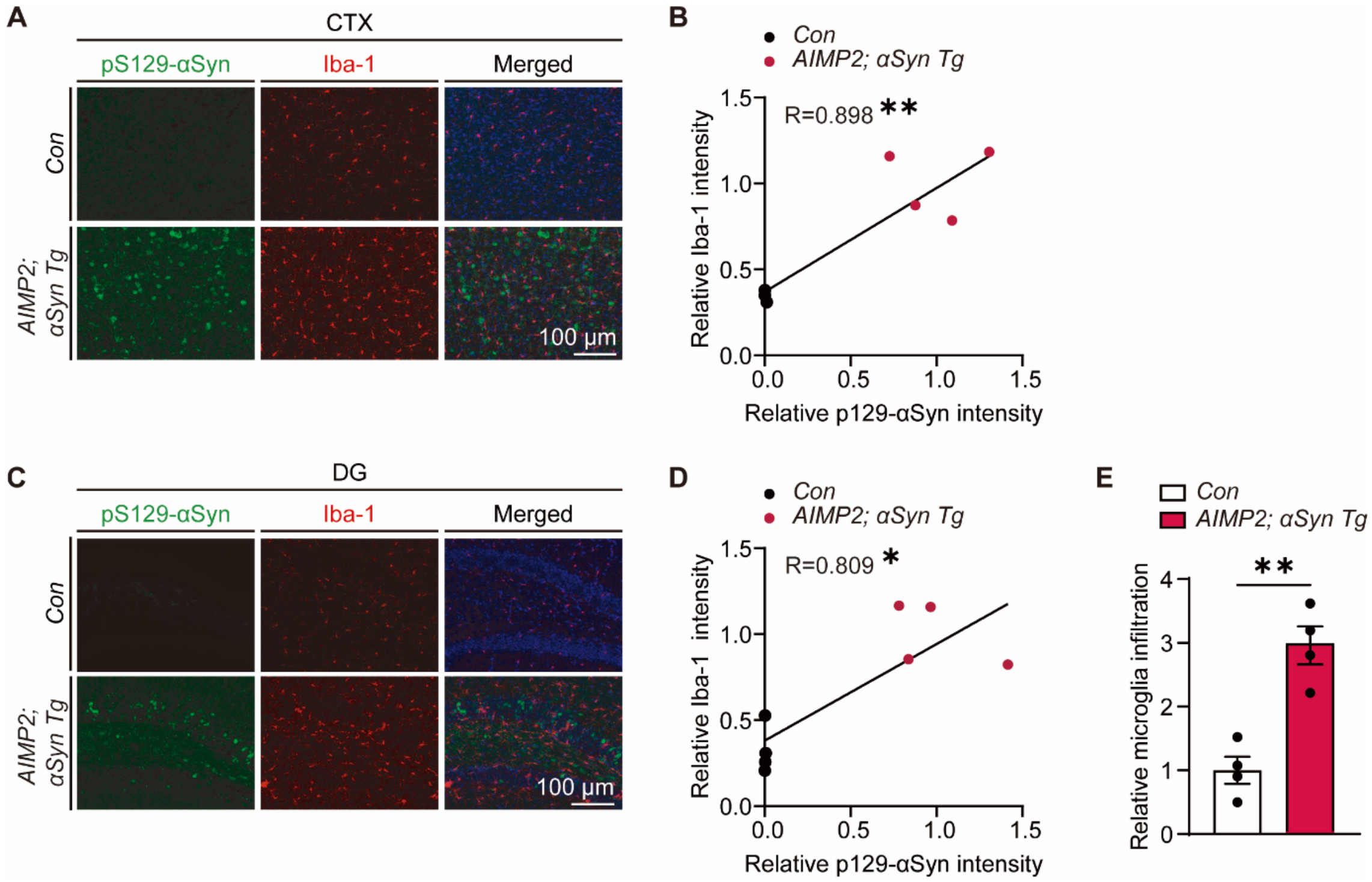

Supplementary figure 3. Microglial activation and infiltration correlate with pS129-αSyn pathology in the cortex and DG of AIMP2;αSyn Tg mice.

(A) Representative immunofluorescence images of Iba-1 and pS129-αSyn in the cortex (CTX) of 2.5 month old AIMP2;αSyn Tg and littermate control. Scale bar = 100 µm.

(B) Pearson correlation dot plot of relative Iba-1 and pS129-αSyn signal intensities with a linear regression line in CTX brain sections from each mouse group (n = 4 mice per group).

(C) Representative immunofluorescence images of Iba-1 and pS129-αSyn in the hippocampal DG of 2.5 month old AIMP2;αSyn Tg and littermate control. Scale bar = 100 µm.

(D) Pearson correlation dot plot of relative Iba-1 and pS129-αSyn signal intensities with a linear regression line in DG brain sections from each mouse group (n = 4 mice per group).

(E) Quantification of Iba-1–positive microglia infiltrating the DAPI-defined, neuron-dense region of the DG in the indicated mouse groups (n = 4 mice per group).

Pearson correlation coefficient (R) was calculated, and statistical significance was assessed using a two-tailed test (B, D). Statistical analysis was performed using a two-tailed Student's *t*-test (E). *$p < 0.05$, and **$p < 0.01$.

1

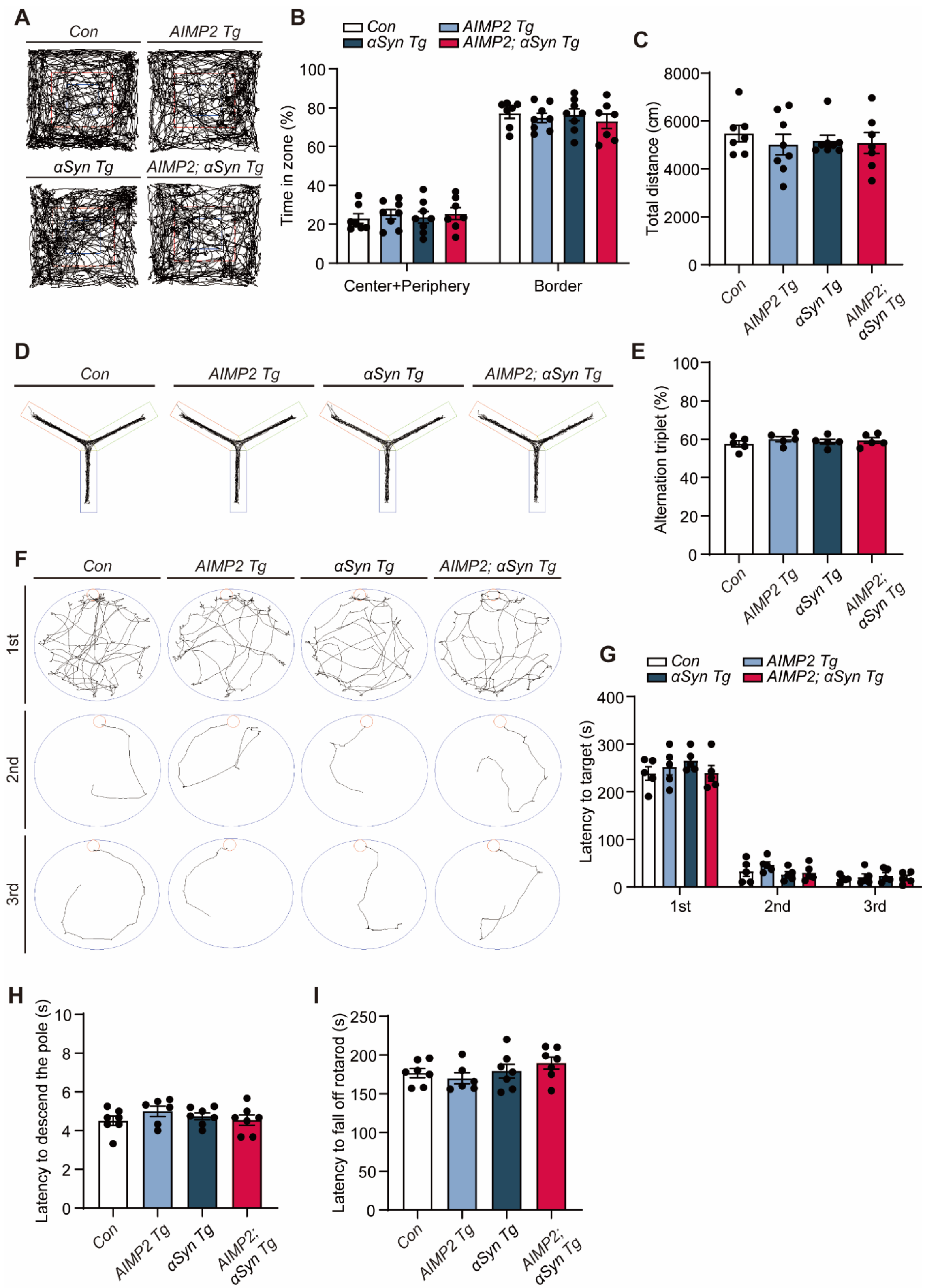

A
Con
AIMP2 Tg
αSyn Tg
AIMP2; αSyn Tg
B
Con
AIMP2 Tg
αSyn Tg
AIMP2; αSyn Tg
Time in zone (%)
Center+Periphery
Border
C
Total distance (cm)
D
E
Alternation triplet (%)
F
1st
2nd
3rd
G
Latency to target (s)
H
Latency to descend the pole (s)
I
Latency to fall off rotarod (s)

Supplementary figure 4. Assessment of baseline behaviors for Tg mice with no transgene expression under doxycycline diet.

(A) Representative exploratory paths of 3 week old mice with the indicated genotypes in an open field arena.

(B) % time spent in the designated regions (border vs. center+periphery) by the indicated mouse groups (*n* = 7 per Con, 8 per AIMP2 Tg or αSyn Tg, 7 per AIMP2;αSyn Tg mice).

(C) Total distance traveled by each mouse group in an open filed test session (*n* = 7 per Con, 8 per AIMP2 Tg or αSyn Tg, 7 per AIMP2;αSyn Tg mice).

(D) Representative travel paths of the indicated 3 week old mouse groups in the Y-maze with three arms.

(E) % spontaneous alteration by each mouse group in the Y-maze (*n* = 5 mice per group).

(F) Representative travel paths by each 3 week old mouse group in three trials (1st-trial on day 1; 2nd-trial on day 3; 3rd-trial on day 8) of Barnes maze. The location of the hole (hiding cage underneath) in the circular platform of the Barnes maze is indicated by red circle.

(G) Latency to reach the target hole in each trial by the indicated mouse groups in the Barnes maze (*n* = 5 mice per group).

(H) Assessment of bradykinesia in the indicated 3 week old mouse groups determined by pole test (*n* = 7 per Con, 6 per AIMP2 Tg, 7 per αSyn Tg or AIMP2;αSyn Tg mice).

(I) Assessment of motor coordination in the indicated 3 week old mouse groups determined by rotarod test (*n* = 7 per Con, 6 per AIMP2 Tg, 7 per αSyn Tg or AIMP2;αSyn Tg mice).

Data are expressed as mean ± SEM. Statistical analysis was performed using a one-way analysis of variance (ANOVA) test followed by Tukey's post-hoc analysis.

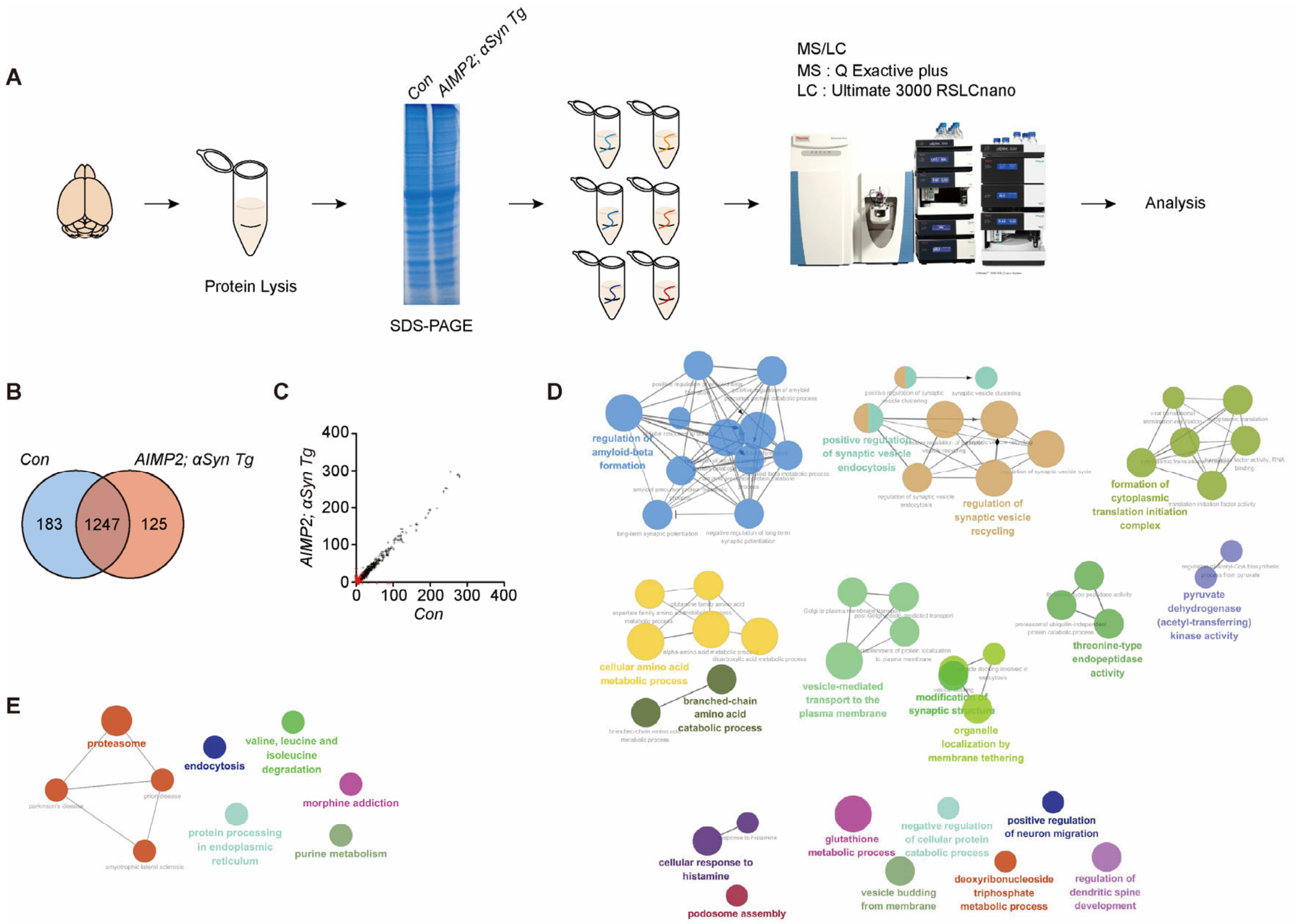

Supplementary figure 5. Identification of differentially expressed proteins in the brains of Lewy body dementia mouse model by mass spectrometry.

(A) Scheme of experimental procedure to isolate total protein lysates from whole brain (except for cerebellum) of control and AIMP2;αSyn Tg mice, followed by SDS-PAGE size separation, and mass spectrometry analysis.

(B) Venn diagram showing the total number of protein identified by mass spectrometry for each mouse brains. Total 1,247 proteins were identified commonly for both genotypes.

(C) Scattor plots of peptide counts of mass spectrometry-identified proteins from total protein lysates of each mouse groups. Proteins with more than 50% changes of peptide counts were indicated as red color.

(D) Functional pathways enriched by differentially expressed proteins identified by mass spectrometry for the whole brains (except for cerebellum) of the 2 month old AIMP2;αSyn Tg mice and littermate control, based on Gene Ontology (GO) biological process analysis, visualized using Cytoscape-ClueGO. Edge thickness indicates

the strength of the functional association between pathway terms.

(E) Functional pathways enriched from differentially expressed proteins identified by mass spectrometry in whole brains (excluding the cerebellum) of 2-month-old AIMP2;αSyn Tg mice and littermate controls, based on KEGG pathway analysis, visualized using Cytoscape-ClueGO. Edge thickness indicates the strength of the functional association between pathway terms.

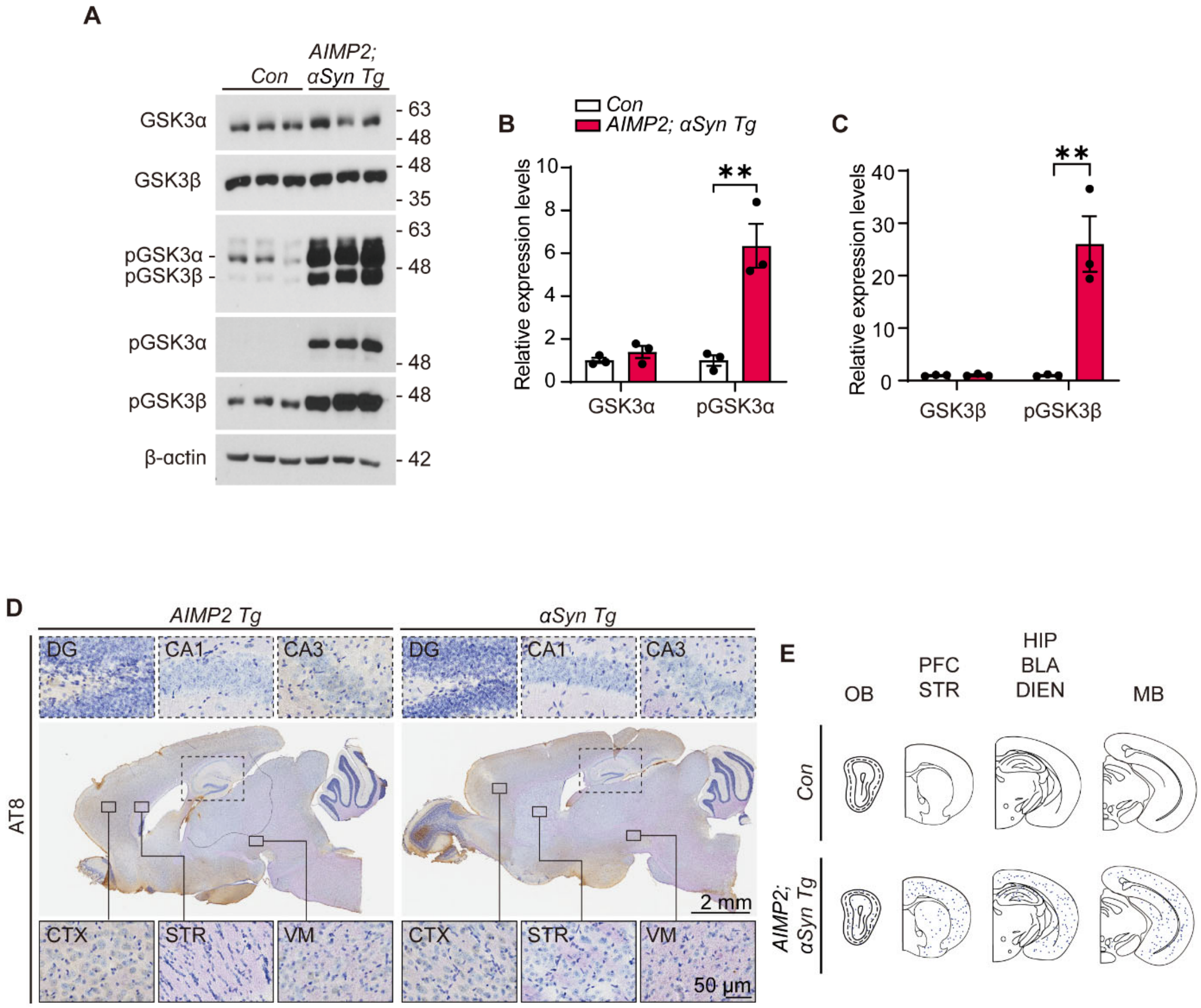

Supplementary figure 6. Assessment of tau-associated signaling pathways and amyloid beta brain pathologies

(A) Representative western blots of glycogen synthase kinase-3α (GSK3α), pS21-GSK3α, GSK3β, and pS9-GSK3β in the whole brain lysates from 2.5 month old AIMP2;αSyn Tg and littermate control mice. β-actin serves as a loading control.

(B, C) Quantification of relative expression of GSK3α, pS21-GSK3α, GSK3β, and pS9-GSK3β in the whole brains from the control and AIMP2; αSyn Tg mice normalized to β-actin. Statistical analysis was performed using a two-tailed Student's t-test. *$p < 0.05$, **$p < 0.01$, and ***$p < 0.001$.

(D) Representative immunohistochemistry of pS202/pT205-Tau (AT8 clone) with Nissl counterstain in the sagittal sections of 2.5 month old AIMP2, and αSyn Tg mice. Magnified images for the selected brain subregions were also shown. Scale bar = 50 μm, and 2 mm as indicated in the images.

(E) Quantitative dot labeling of phosphorylated tau expression in each brain regions of 2.5 month old AIMP2;αSyn Tg and littermate control integrated by AT8 immunohistochemistry image analysis.

Data are expressed as mean ± SEM. Statistical analysis was performed using a two-tailed Student's *t*-test. $^{*}p < 0.05$, $^{**}p < 0.01$, and $^{***}p < 0.001$. The uncropped Western blot panels are provided in Supplementary figure 10.

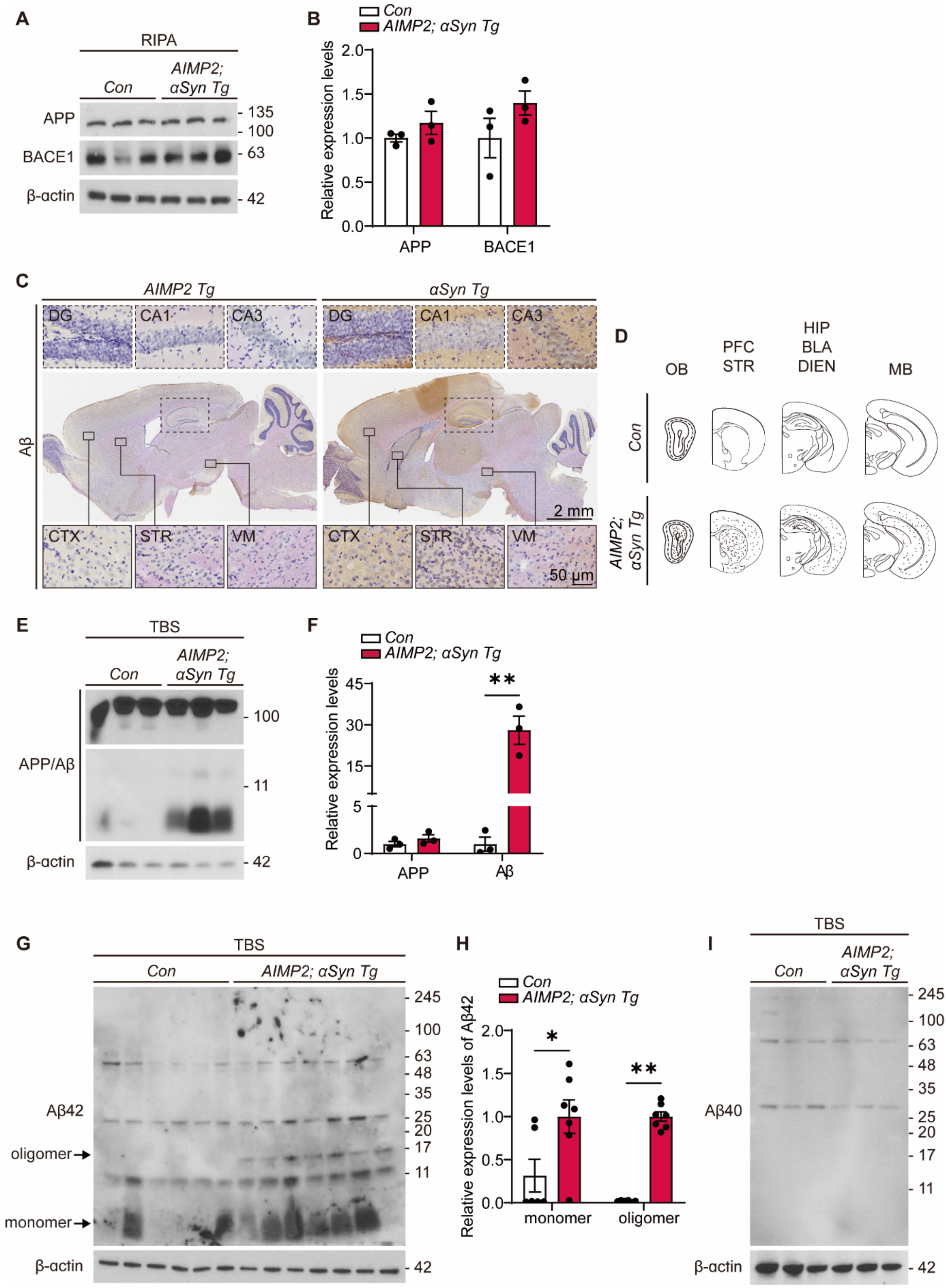
A
RIPA
Con
AIMP2; αSyn Tg
APP
BACE1
β-actin
135
100
63
42
B
Con
AIMP2; αSyn Tg
Relative expression levels
APP
BACE1
C
AIMP2 Tg
αSyn Tg
DG
CA1
CA3
Aβ
CTX
STR
VM
2 mm
50 µm
D
OB
PFC
STR
HIP
BLA
DIEN
MB
Con
AIMP2; αSyn Tg
E
TBS
APP/Aβ
β-actin
F
Relative expression levels
APP
Aβ
**
G
Aβ42
oligomer
monomer
H
Relative expression levels of Aβ42
monomer
oligomer
*
**
I
Aβ40
245
100
63
48
35
25
20
17
11

Supplementary figure 7. Elevated Aβ42 accumulation and soluble Aβ42 oligomeric species in AIMP2;αSyn Tg brains without changes in APP and BACE expression.

(A) Representative western blots of amyloid precursor protein (APP) and β-site APP–cleaving enzyme (BACE) in whole-brain RIPA lysates from 2.5-month-old AIMP2;αSyn Tg and littermate control mice. β-actin was used as a loading control.

(B) Quantification of APP and BACE1 expression levels in whole-brain lysates from control and AIMP2;αSyn Tg mice, normalized to β-actin (*n* = 3 mice per group).

(C) Representative immunohistochemistry of amyloid beta with Nissl counterstain in the sagittal sections of 2.5 month old AIMP2, and αSyn Tg mice. Magnified images for the selected brain subregions were also shown. Scale bar = 50 um, and 2 mm as indicated in the images.

(D) Quantitative dot labeling of amyloid beta expression in each brain regions of 2.5 month old AIMP2;αSyn Tg and littermate control integrated by anti-amyloid beta immunohistochemistry image analysis.

(E) Representative western blots of APP/Aβ in whole-brain Tris-buffered saline (TBS) lysates from 2.5-month-old AIMP2;αSyn Tg and littermate control mice. Aβ blot was acquired with longer exposure than the APP blot. β-actin was used as a loading control.

(F) Quantification of APP and Aβ expression levels in whole-brain lysates from control and AIMP2;αSyn Tg mice, normalized to β-actin (*n* = 3 mice per group).

(G) Representative western blots of amyloid-β 42 (Aβ42) and its oligomeric forms in whole-brain TBS lysates from 2.5-month-old AIMP2;αSyn Tg and littermate control mice. β-actin was used as a loading control.

(H) Quantification of Aβ42 and its oligomeric forms in whole-brain lysates from control and AIMP2;αSyn Tg mice, normalized to β-actin (*n* = Con 6, AIMP2;αSyn Tg 7 mice).

(I) Representative western blots of amyloid-β 40 (Aβ40) in whole-brain TBS lysates from 2.5-month-old AIMP2;αSyn Tg and littermate control mice. β-actin was used as a loading control.

Data are expressed as mean ± SEM. Statistical analysis was performed using a two-tailed Student's *t*-test. (B, F) and the Mann–Whitney test (H). $*p < 0.05$, $**p < 0.01$. The uncropped Western blot panels are provided in Supplementary figure 10.

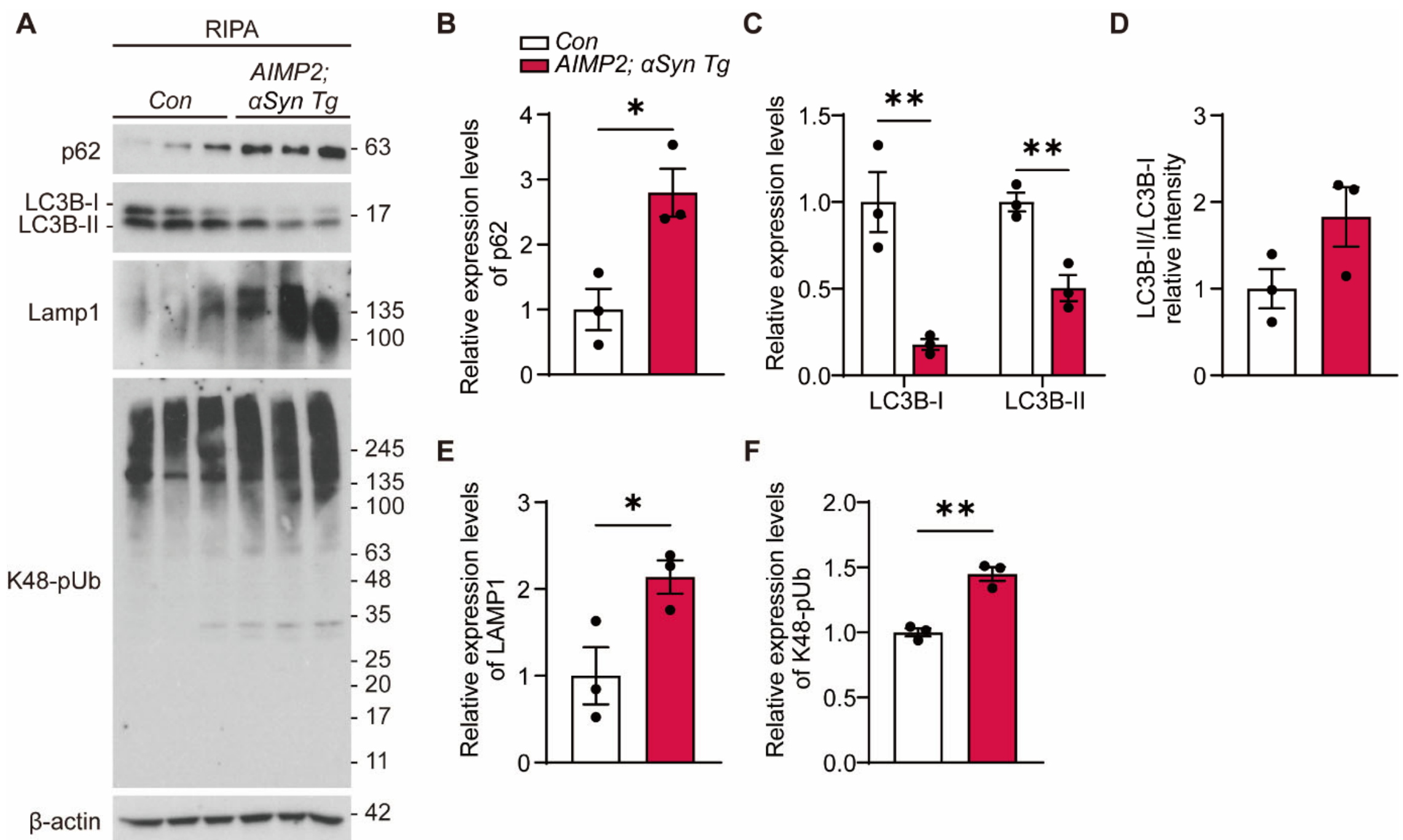

Supplementary figure 8. Impaired autophagic flux and proteasomal dysfunction-associated changes in AIMP2;αSyn Tg mouse brains.

(A) Representative western blots of p62, microtubule-associated protein 1 light chain 3 beta-I (LC3B-I), LC3B-II, Lamp1, and K48-linked polyubiquitin chains (K48-pUb) in brain RIPA lysates from 2.5-month-old AIMP2;αSyn Tg and littermate control mice. β-actin was used as a loading control.

(B, C, D, E, and F) Quantification of p62, LC3B-I, LC3B-II, LC3B-II/I ratio, Lamp1, and K48-pUb in brain lysates from control and AIMP2;αSyn Tg mice, normalized to β-actin (n = 3 mice per group).

Data are expressed as mean ± SEM. Statistical analysis was performed using a two-tailed Student's t-test. *$p < 0.05$, **$p < 0.01$. The uncropped Western blot panels are provided in Supplementary figure 10.

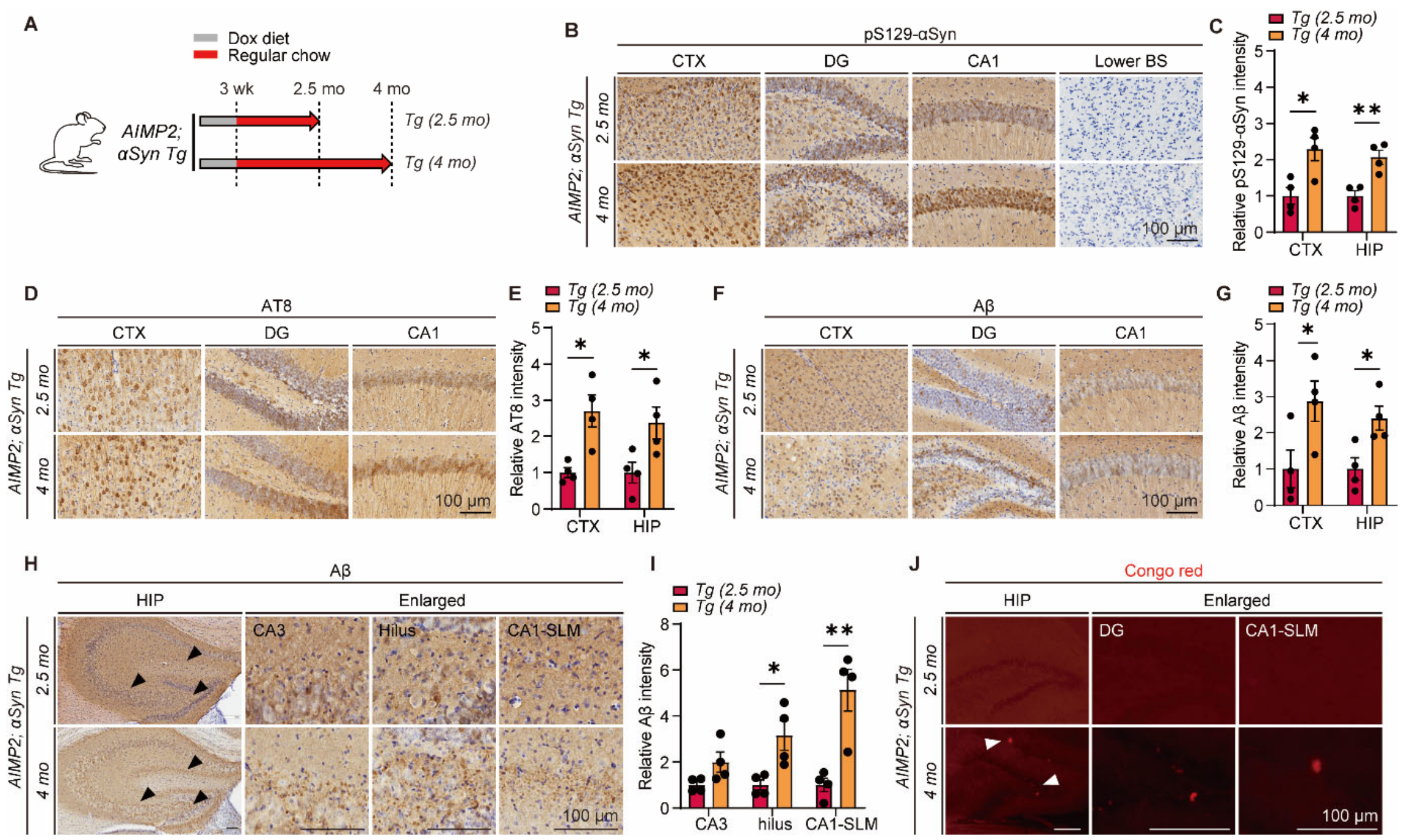

Supplementary figure 9. Progressive accumulation of pS129-αSyn, AT8-positive Tau, and amyloid-β pathologies in the cortex and hippocampus of AIMP2;αSyn Tg mice.

(A) Schematic diagram of the experimental timeline for prolonged induction of AIMP2 and αSyn in Tg mice, including timed administration of doxycycline-containing diet or regular chow.

(B) Representative immunohistochemistry images of αSyn aggregates in the indicated brain subregions from 2.5- and 4-month-old AIMP2;αSyn Tg mice using pS129-αSyn specific antibody. The sections were counterstained with Nissl. Scale bar = 100 μm. cortex, CTX; Dentate gyrus, DG; Cornu Ammonis-1, CA1, brainstem; BS.

(C) Quantification of pS129-αSyn signals in the CTX and HIP (DG + CA1) from 2.5- and 4-month-old AIMP2;αSyn Tg mice (n = 4 mice per group).

(D) Representative immunohistochemistry of pS202/pT205-Tau (AT8 clone) with Nissl counterstain in the indicated brain subregions from 2.5- and 4-month-old AIMP2;αSyn Tg mice. Scale bar = 100 μm.

(E) Quantification of AT8 signals in the CTX, and HIP (DG + CA1) from 2.5- and 4-month-old AIMP2;αSyn Tg mice (n = 4 mice per group).

(F) Representative immunohistochemistry of amyloid beta with Nissl counterstain in the indicated brain subregions from 2.5- and 4-month-old AIMP2;αSyn Tg mice. Scale bar = 100 μm.

(G) Quantification of amyloid beta signals in the CTX, and HIP (DG + CA1) of each mouse group (n = 4 mice per group).

(H) Extracellular and intracellular distribution of amyloid beta in those indicated brain subregions (CA3, hilus, and CA1-SLM) of 2.5- and 4-month-old AIMP2;αSyn Tg mice monitored by anti-Aβ immunohistochemistry. Scale bar = 100 μm. CA1-SLM, the stratum lacunosum-moleculare of the CA1 region.

(I) Quantification of amyloid beta signal intensities in the indicated brain subregions of 2.5- and 4-month-old AIMP2;αSyn Tg mice (n = 4 mice per group).

(J) Representative Congo red–stained hippocampal sections from 2.5- and 4-month-old AIMP2;αSyn Tg mice. Higher-magnification images are shown in the inset panels.

Data are expressed as mean ± SEM. Statistical analysis was performed using a two-tailed Student's t-test. *$p < 0.05$, **$p < 0.01$.

Fig1B

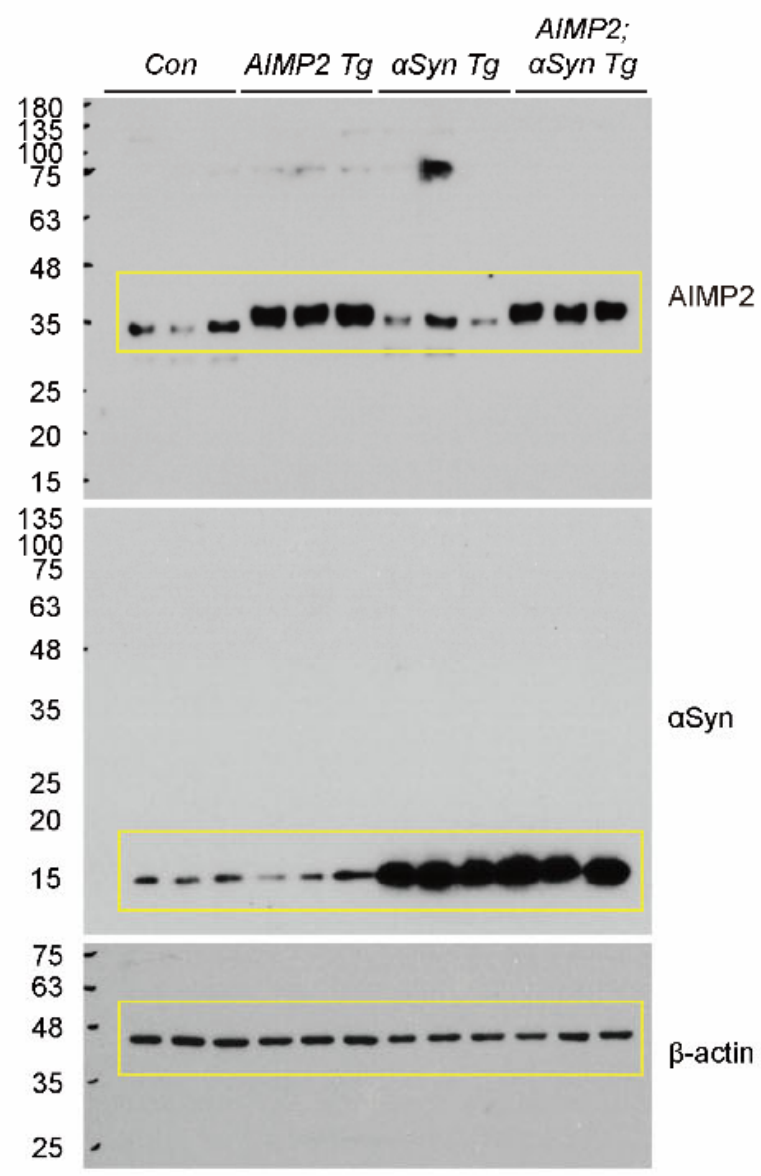

Fig2C

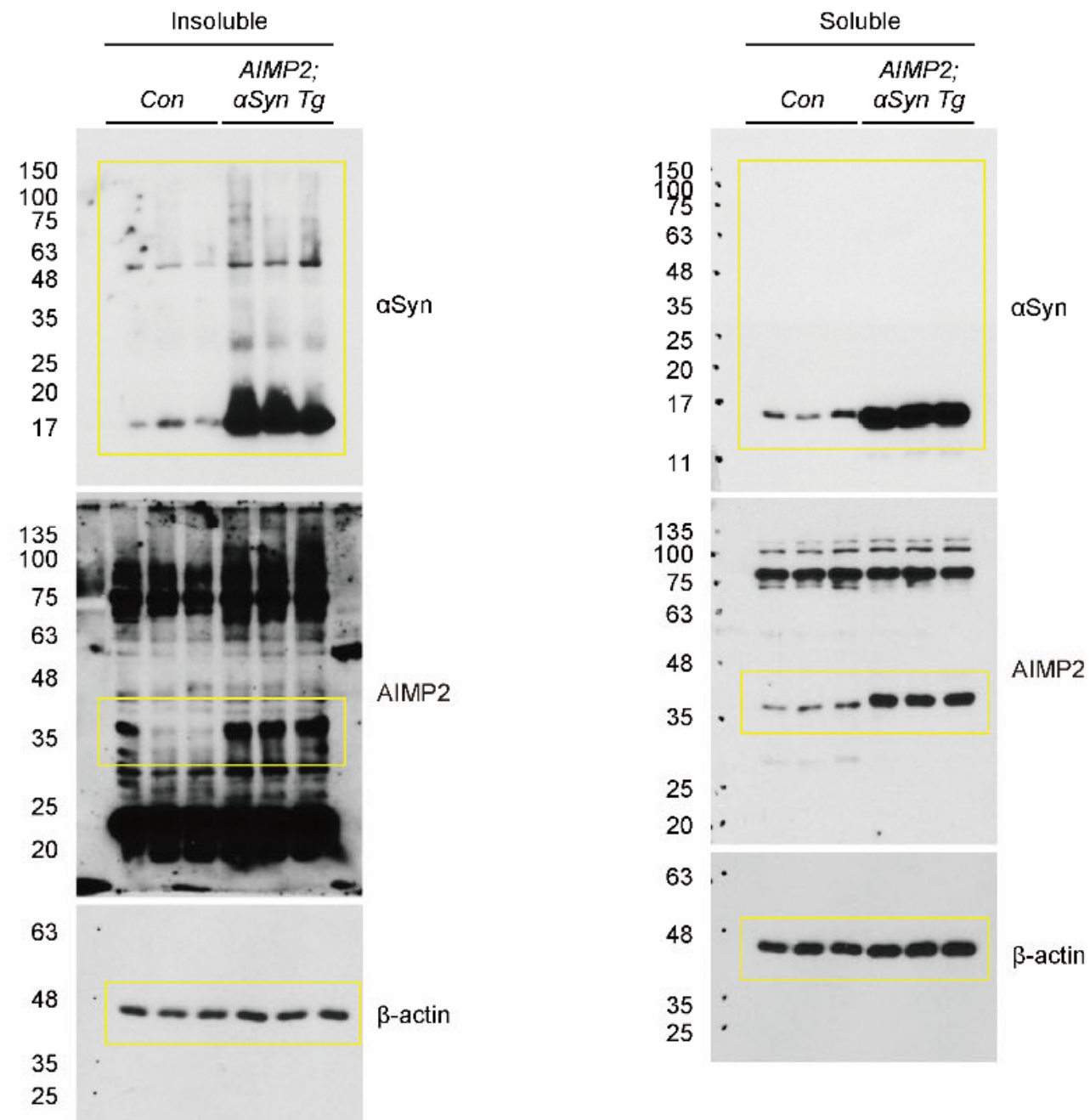

Fig5A

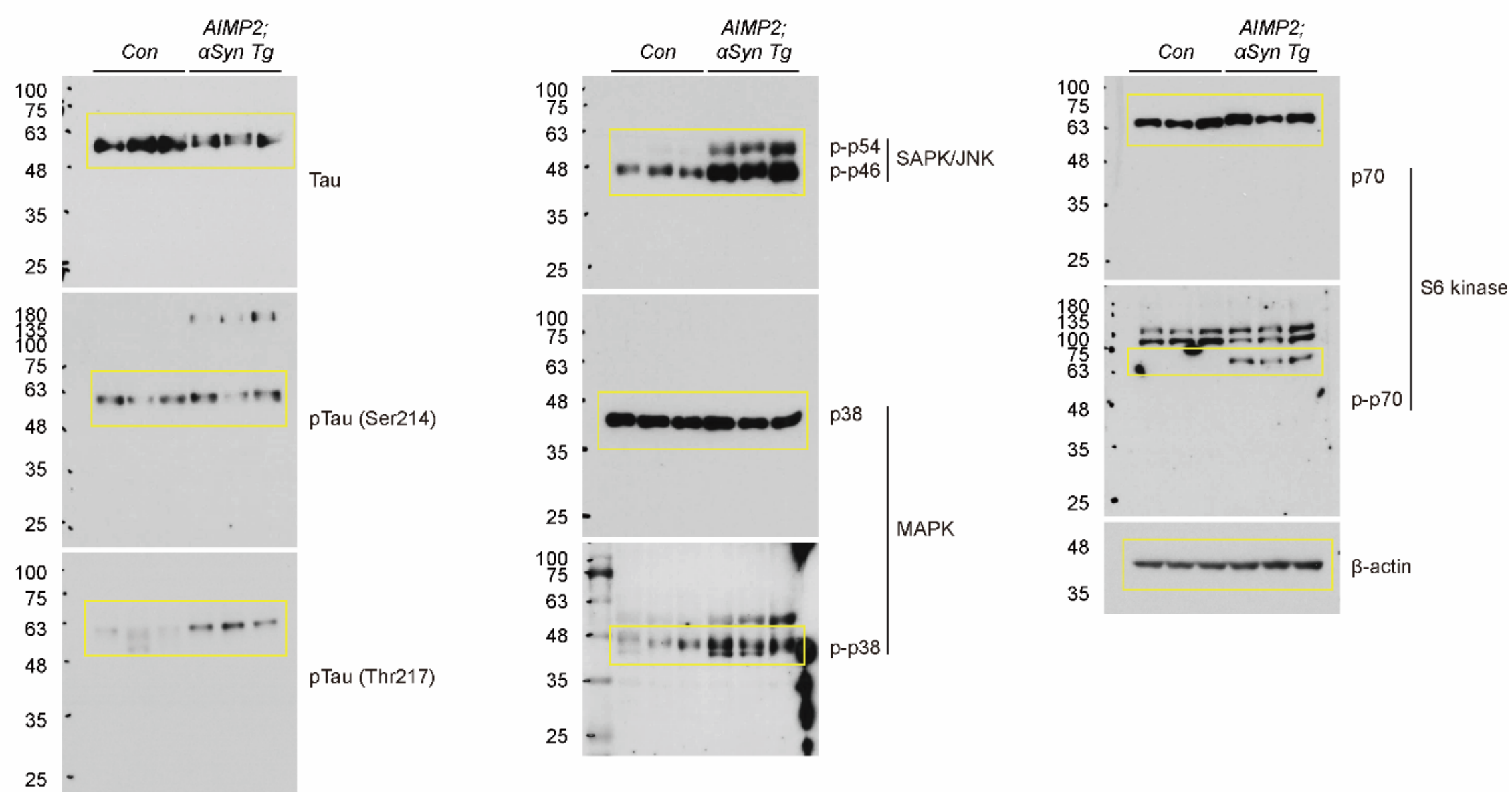

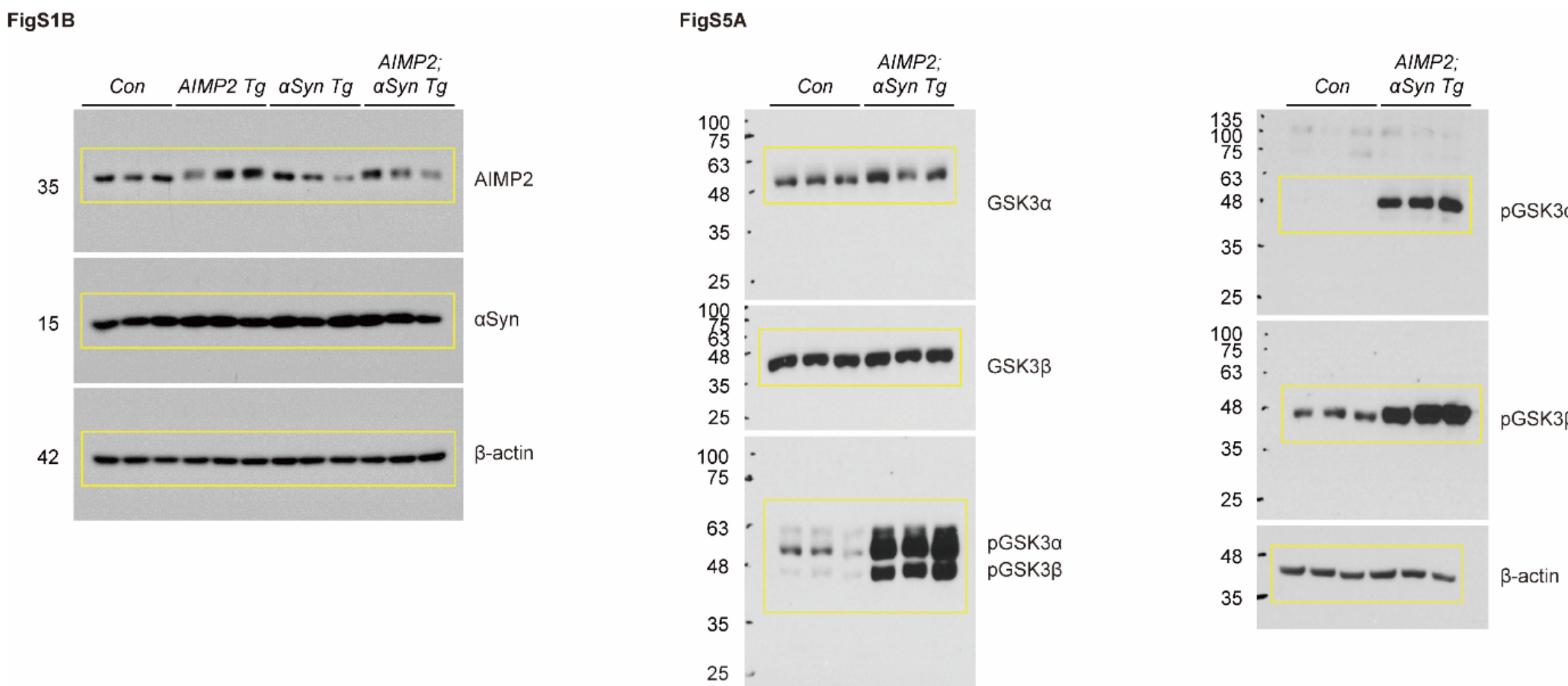

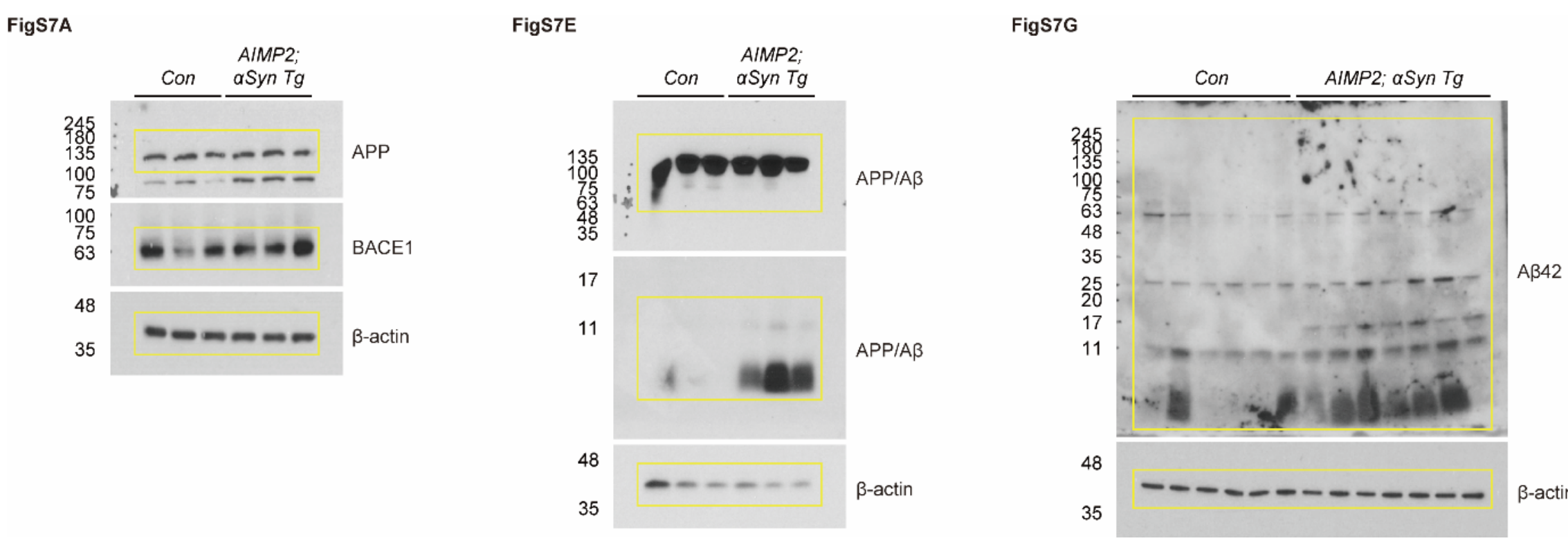

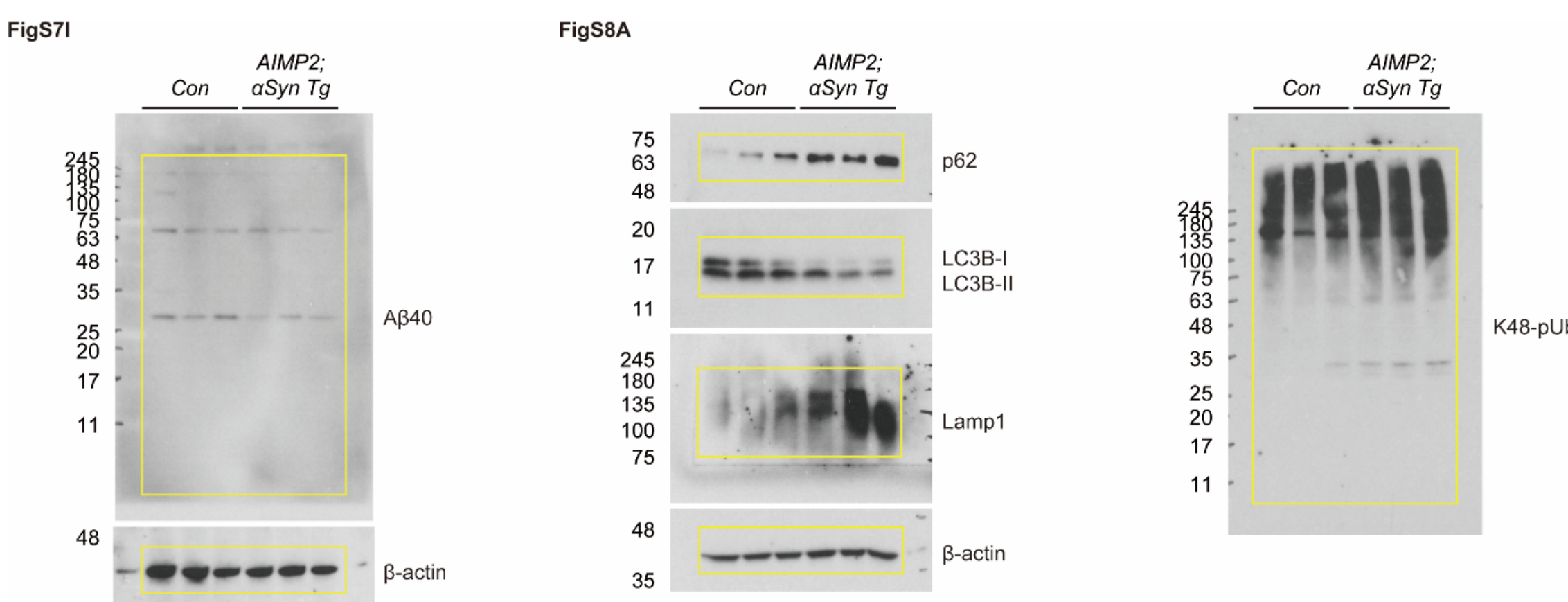

Supplementary figure 10. Uncropped Western blot images

Full, uncropped Western blot panels corresponding to those shown in the main and supplementary figures are provided with figure panel labels. Molecular weight markers are indicated, and the regions included in the main figures are boxed.

Supplementary table 1. List of proteins identified in the whole brain lysates of mice by mass spectrometry.

Supplementary table 2. Functional pathway analysis (GO-Biological Processes) for differentially expressed proteins.

Supplementary table 3. Functional pathway analysis (KEGG) for differentially expressed proteins.

Supplementary table 4. Endeavour gene prioritization for the differentially expressed proteins.

References

1. Bindea G, Mlecnik B, Hackl H, Charoentong P, Tosolini M, Kirilovsky A*, et al.* ClueGO: a Cytoscape plug-in to decipher functionally grouped gene ontology and pathway annotation networks. *Bioinformatics* 2009, **25**(8)**:** 1091-1093.

2. Tranchevent LC, Ardeshirdavani A, ElShal S, Alcaide D, Aerts J, Auboeuf D*, et al.* Candidate gene prioritization with Endeavour. *Nucleic Acids Res* 2016, **44**(W1)**:** W117-121.
