# Supplementary material for: Neuronal AIMP2–α-synuclein synergy drives endogenous tau and amyloid-β pathologies and neurodegeneration in a mechanistic model of Lewy body dementia
Source: Cell Death Dis. 2026 Aug 13;17(1):959. doi: 10.1038/s41419-026-09181-3 (PMC13752285; doi:10.1038/s41419-026-09181-3)
Supplement: Supplementary file 2 [file 41419_2026_9181_MOESM2_ESM.pdf]

Fig1B

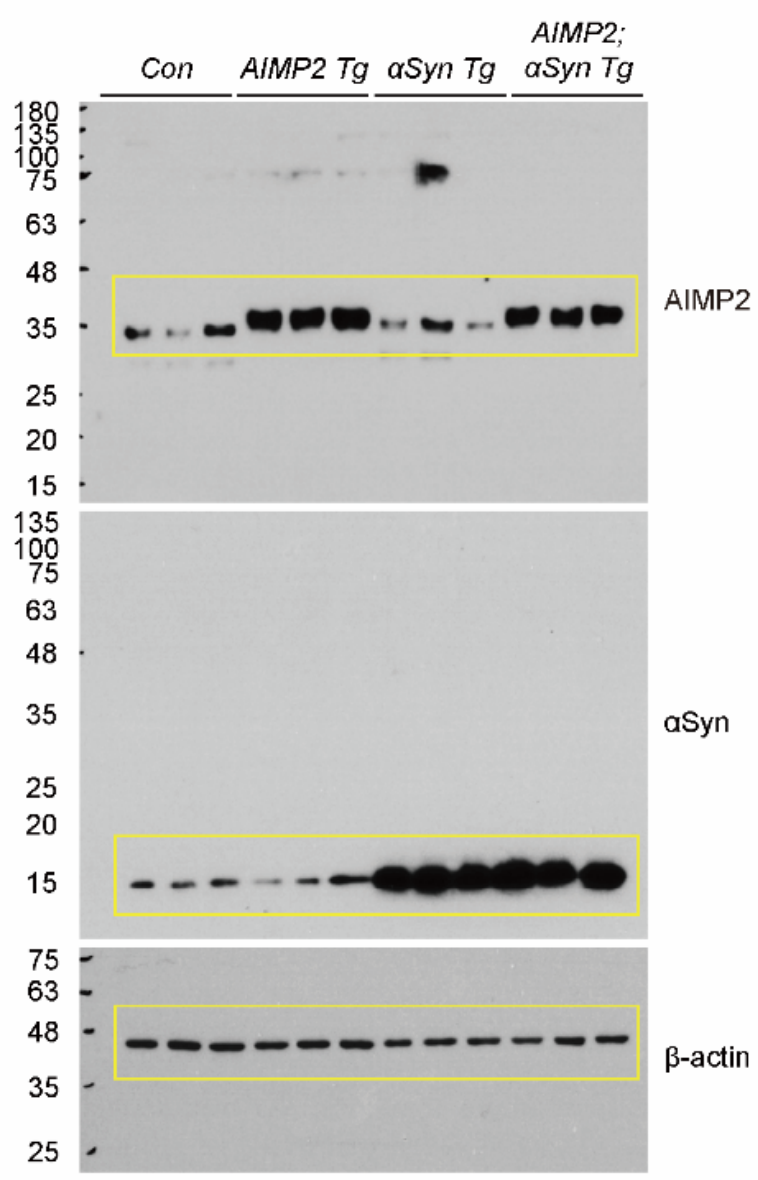

Fig2C

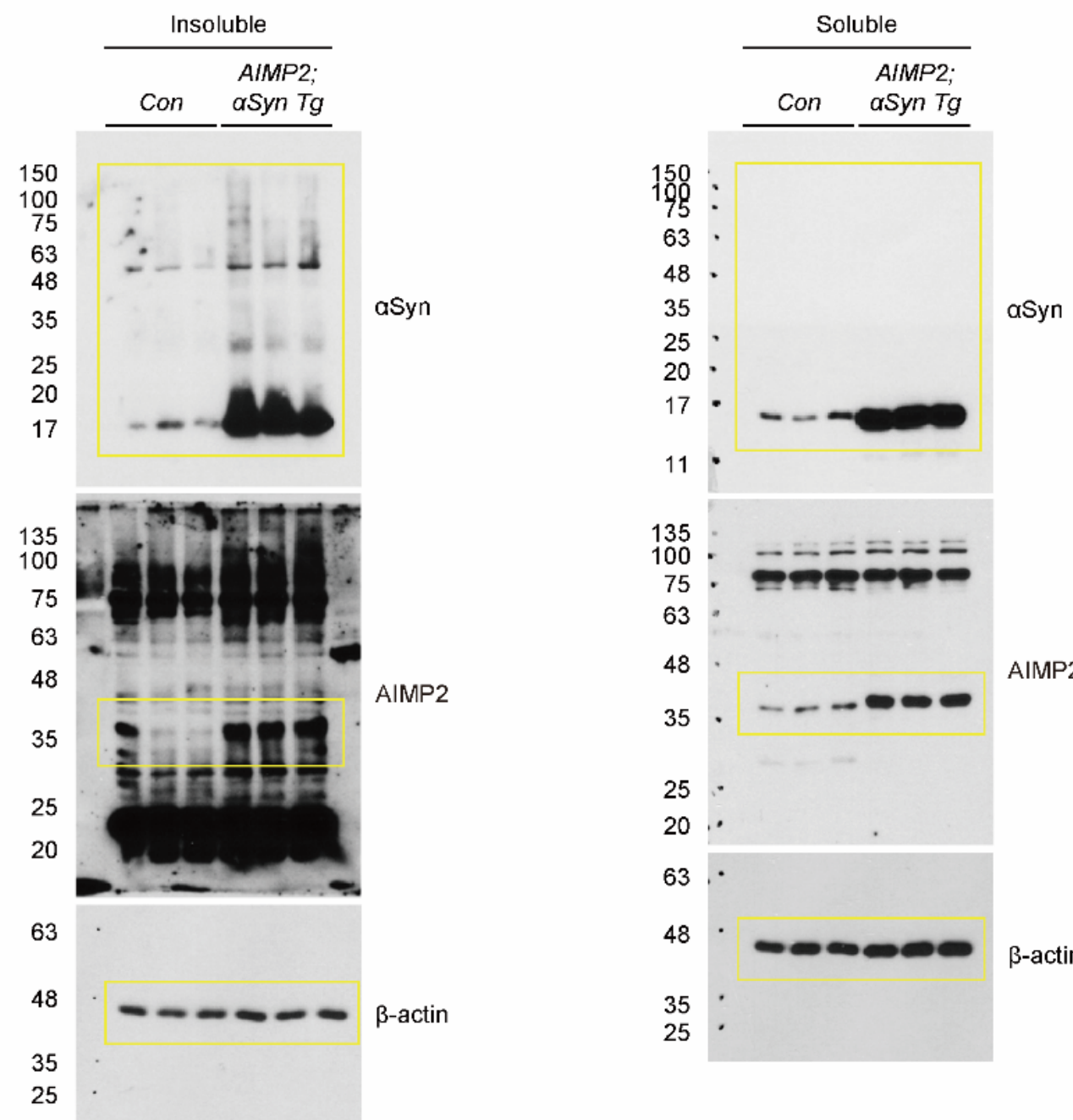

Fig5A

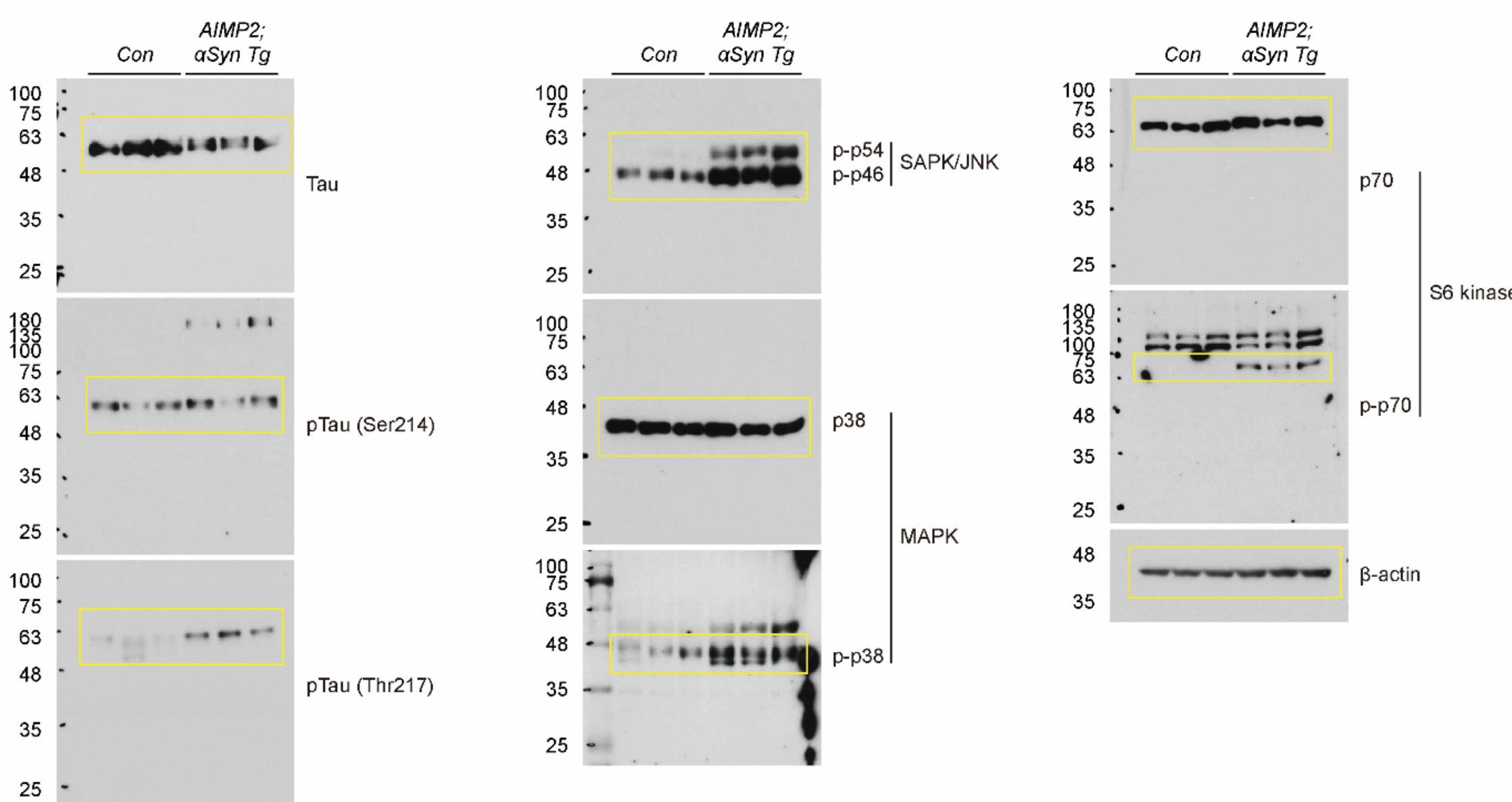

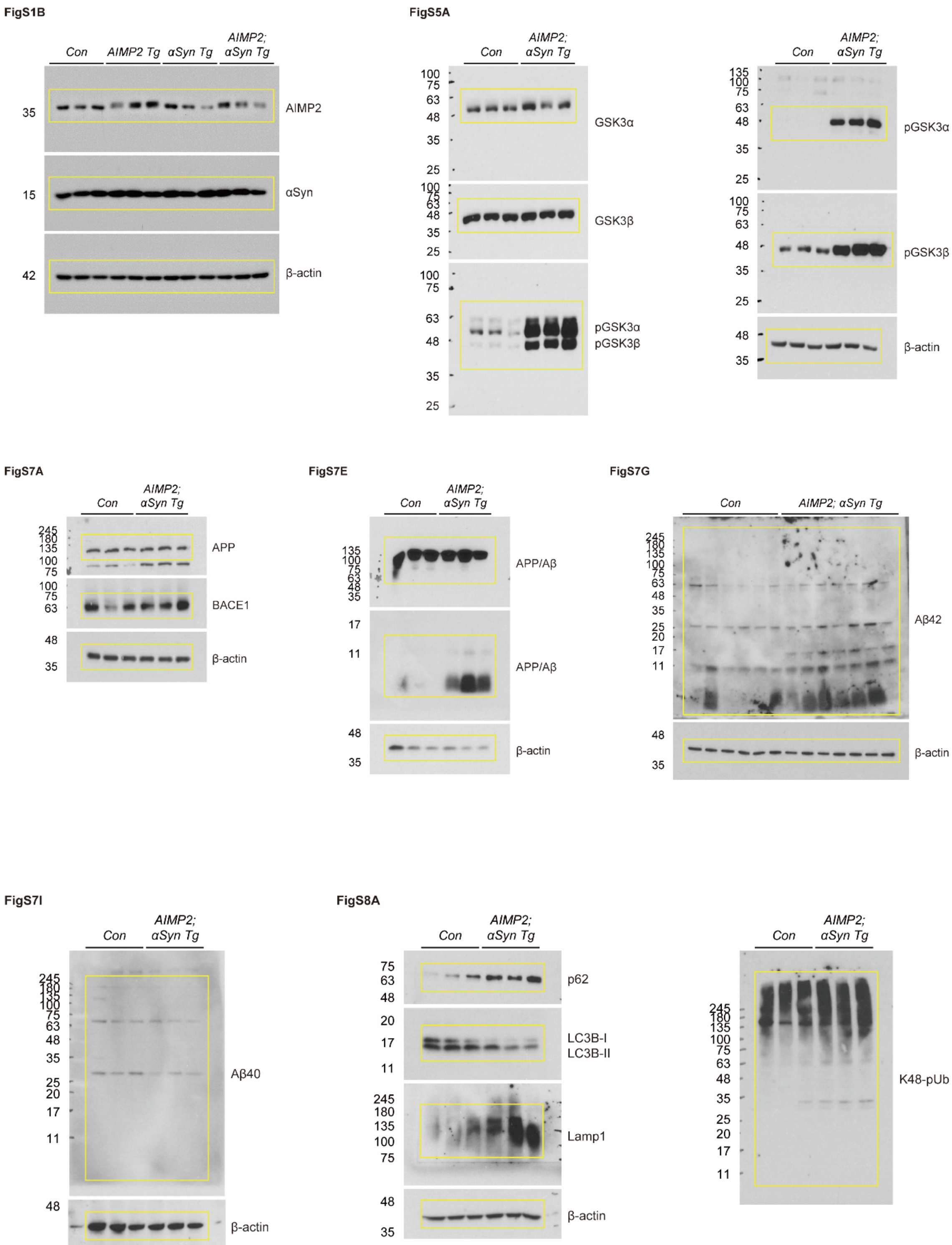

Supplementary figure 10. Uncropped Western blot images

Full, uncropped Western blot panels corresponding to those shown in the main and supplementary figures are provided with figure panel labels. Molecular weight markers are indicated, and the regions included in the main

figures are boxed.
